# Supplementary material for: A reprogrammable mechanical metamaterial with origami functional-group transformation and ring reconfiguration
Source: Nat Commun. 2023 Oct 23;14:6709. doi: 10.1038/s41467-023-42323-1 (PMC10593812; doi:10.1038/s41467-023-42323-1)
Supplement: Supplementary file 1 — Supplementary Information [file 41467_2023_42323_MOESM1_ESM.pdf]

Supplementary Material

for

"*A reprogrammable mechanical metamaterial with origami functional-group transformation and ring reconfiguration*"

Xinyu Hu, Ting Tan, Benlong Wang, Zhimiao Yan*

* correspondence to: zhimiaoy@sjtu.edu.cn (Zhimiao Yan)

This file includes:

Supplementary Discussions 1 to 8

Supplementary Figures 1 to 44

Supplementary Tables 1 to 3

Supplementary References

1. Basic design and relevant parameters

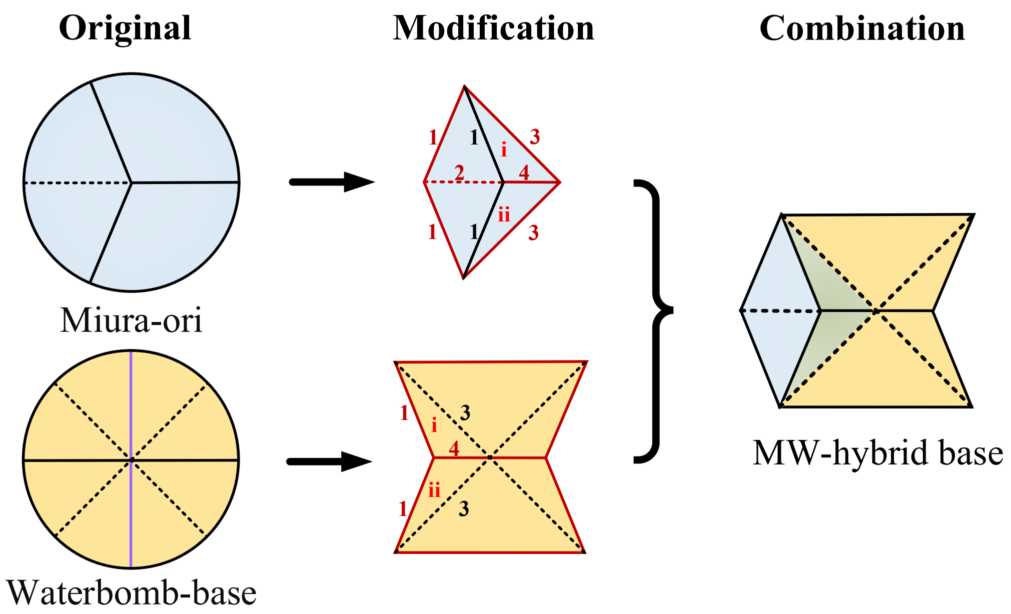

Supplementary Fig. 1: **Schematic of the design method of MW-hybrid base for the origami construction**. The solid and dashed lines represent the mountain and valley creases, respectively.

In the modified Miura-ori and waterbomb-base elements, as shown in Supplementary Fig. 1, the black and red lines represent the original and modified creases, respectively. Black crease 1 of the Miura-ori element is taken as the waist of the isosceles triangle to determine the other waist line, i.e., red crease 1. Red crease 2 is then determined as the bottom edge of the isosceles triangle in Miura-ori element. For element fusion, black crease 1 of the Miura-ori element and black crease 3 of the waterbomb-base element are used to determine red crease 1 of the waterbomb-base element and red crease 3 of the Miura-ori element, respectively, as the two creases with the same number should coincide. With crease 1 and crease 3 determined, red crease 4 of the two elements is obtained via a triangle relationship. The vertical mountain crease (purple line) of the original waterbomb-base element is removed to reduce the structural degrees of freedom. After fusion by superimposing facets i and facets ii, the MW-hybrid base is designed as the element of the origami. The remaining mountain and valley crease configurations of the original Miura-ori and waterbomb elements are reserved in the hybrid element.

The folding properties of the Miura-ori and waterbomb-base elements are, respectively, determined by the plane angles α and $\beta/2$. Therefore, the folding perpoty of origami constructed by the modification

and fusion of the Miura-ori and waterbomb-base elements is also affected by parameters α and β, as shown in Supplementary Fig. 2(a). To avoid excessive geometric interference between non-adjacent

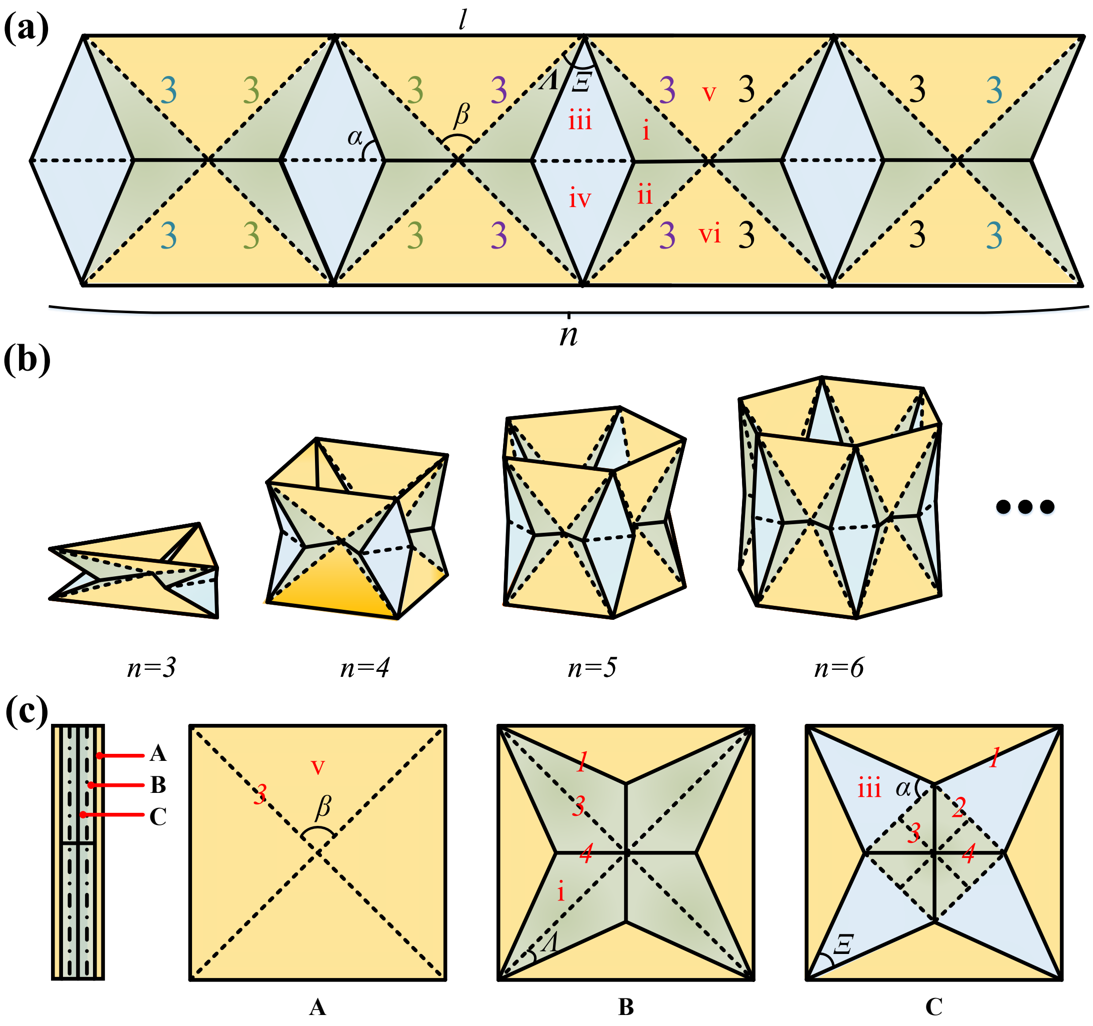

Supplementary Fig. 2: **The schematic diagram of origami**. (a) The crease pattern of origami with n = 4. (b) Three-dimensional diagrams of the origami for n = 3, 4, 5 and 6 (from left to right) at maximum folding height H_{max}. (c) The side view of origami with n = 4 in a flat folded state and the top view of the folded layers A, B and C.

surfaces but maintain a compact folding effect, valley creases 3 sharing the same vertex are designed to coincide completely when the interweaved origami is flat folded, as shown in Supplementary Fig. 2(c).

Six folded layers exist when the origami is in a flat folded state, as shown in Supplementary Fig. 2(c). Three types of folded layers A, B and C are symmetrical along the height. From the top view of folded layer A, the side number n and static plane angle β are noted to have the relationship $\beta = 2\pi/n$.

Based on the geometric relationships shown in folded surfaces B and C, the angle Ξ in facet iii is twice as much as the angle Λ in facet i. Inspecting Supplementary Fig. 2(a), it can also be determined that $\Lambda = \beta/2 + \alpha - \pi/2$ and $\Xi = \pi - 2\alpha$. Therefore, the relationship between static plane angles β and α is determined to be $\beta = 2(\pi - 2\alpha)$. In addition, the lengths of crease 1, crease 2, crease 3 and crease 4 can be calculated as

$$
\begin{aligned}
l_1 &= \frac{l}{2\tan(\beta/2)\sin\alpha} \\
l_2 &= \frac{l}{\tan(\beta/2)\tan\alpha} \\
l_3 &= \frac{l}{2\sin(\beta/2)} \\
l_4 &= \frac{l}{2} - \frac{l}{2\tan(\beta/2)\tan\alpha}
\end{aligned}
\qquad (1)
$$

2. Position analysis

Generally, the mechanical properties of origami are greatly affected by the structural folding procedure. As such, the structural kinematics of origami are first investigated by spherical trigonometry. Since the facets' deformation is very small during folding, the approximate rigid foldable hypothesis is introduced to determine dihedral angles as functions of the origami height. The modified Miura-ori and Waterbomb-base elements are separately analysed with spherical mechanisms to realise this purpose.

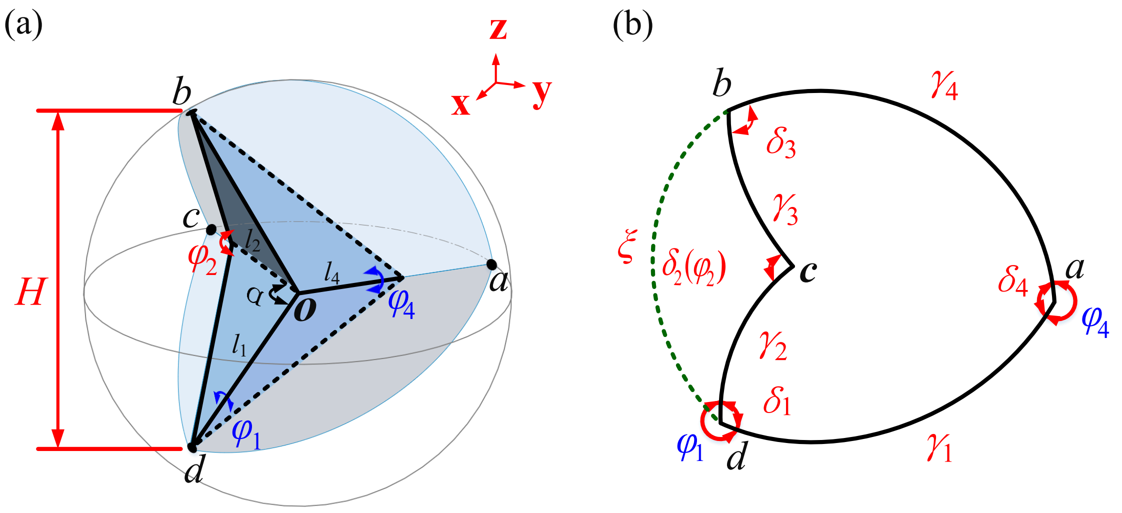

Supplementary Fig. 3: The (a) 3D and (b) 2D representations of spherical trigonometry for modified Miura-ori elements.

Supplementary Fig. 3(a) and (b) present the 3D and 2D spherical trigonometry of modified Miura-ori elements in unit spheres, respectively. γ_i(i=1,2,3,4) in Supplementary Fig. 3(b) represents the arc length ($\widehat{ad}$, $\widehat{cd}$, $\widehat{bc}$, $\widehat{ab}$) in Supplementary Fig. 3(a). δ_i denotes the dihedral angle between intersecting facets. Inspecting Supplementary Fig. 2(a) and Supplementary Fig. 3, it can be obtained that $\gamma_1 = \gamma_4 = \pi - \alpha$, $\gamma_2 = \gamma_3 = \alpha$, $\delta_1 = \delta_3 = 2\pi - \varphi_1$, $\delta_2 = \varphi_2$ and $\delta_4 = 2\pi - \varphi_4$. The dihedral angles φ_1 and φ_4 are the dihedral angles at the mountain creases, and the dihedral angle φ_2 denotes the dihedral angle at the valley crease. Based on the relationships between the dihedral angles of a degree−4 flat vertex fold [1], the dihedral angles φ_1 and φ_4 can be expressed as a function of dihedral angle φ_2 and static plane angle α:

$$\begin{cases} \varphi_1 = \pi + 2\cos^{-1}\dfrac{\cos\alpha - \cos\xi\cos\alpha}{\sin\xi\sin\alpha} \\ \varphi_4 = 2\pi - \varphi_2 \end{cases} \tag{2}$$

where ξ is the radian value of the auxiliary side in spherical $\triangle abd$ and spherical $\triangle bcd$, which is given by $\xi = \cos^{-1}(\cos^2\alpha + \sin^2\alpha\cos\varphi_2)$. Using the Pythagorean theorem, the relationship between height H and dihedral angle φ_2 can be obtained as

$$\frac{l}{2\tan(\beta/2)}\sin\frac{\varphi_2}{2} = \frac{H}{2} \tag{3}$$

Thus, the dihedral angle φ_2 can be determined as a function of the structural height H:

$$\varphi_2 = 2\sin^{-1}\left[\frac{H\tan(\beta/2)}{l}\right] \tag{4}$$

Supplementary Fig. 4(a) and (b) present the modified Waterbomb-base element's 3D and 2D spherical trigonometry in a unit sphere, respectively. Due to the symmetric property [2], there are only two types of dihedral angles, φ_3 and φ_4, among the 6 creases in the modified Waterbomb-base element. The dihedral angle φ_4 denotes the motion of the mountain crease and exists in the modified Miura-ori cell. The dihedral angle φ_3 presents the motion of the valley crease. To determine the valley dihedral angle φ_3, facets ofh and oeh are introduced as auxiliary sides to form the complete spherical $\triangle gfh$ and spherical $\triangle efh$. In spherical $\triangle gfh$, λ_1, λ_2 and ω are arc lengths of $\widehat{fg}$, $\widehat{gh}$ and $\widehat{fh}$, respectively. Inspecting Supplementary Fig. 2(a) and Supplementary Fig. 4(a), it is obtained that λ_1 = λ_2 = (π-β)/2. τ denotes the dihedral angle between facet ofg and facet ofh. The relationship between arc length ω and dihedral

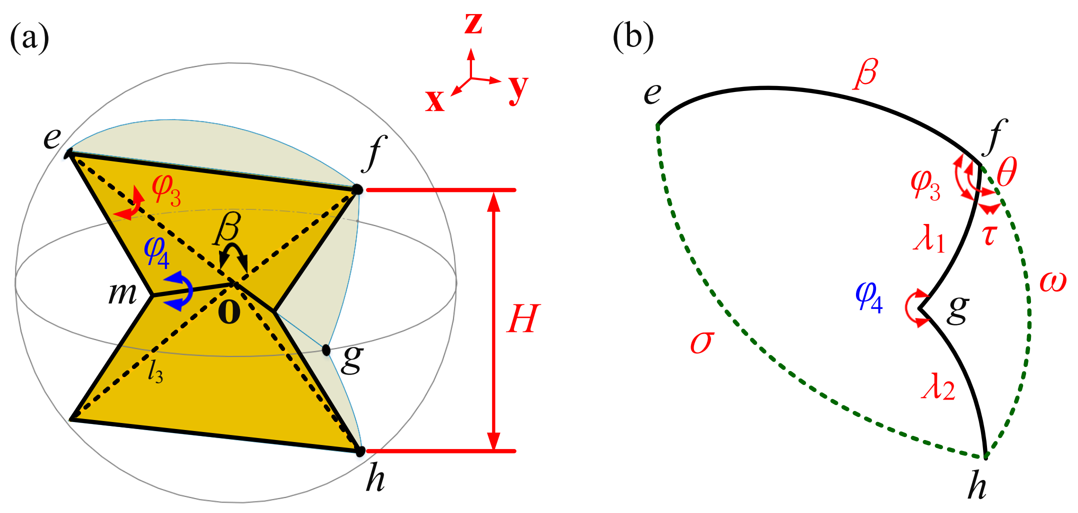

Supplementary Fig. 4: **The (a) 3D and (b) 2D representations of spherical trigonometry for modified Waterbomb-base elements**

angle φ_4 is determined by the cosine theorem of spherical trigonometry in spherical $\triangle gfh$ as

$$\cos\omega = \cos(\lambda_1)\cos(\lambda_2) + \sin(\lambda_1)\sin(\lambda_2)\cos(2\pi - \varphi_4) \tag{5}$$

Based on Supplementary Equations 2 and 5, the arc length ω is calculated as

$$\omega = \cos^{-1}\left(\sin^2\frac{\beta}{2} + \cos^2\frac{\beta}{2}\cos\varphi_2\right) \tag{6}$$

Moreover, the relationship between arc length ω and dihedral angle τ is determined by the sine theorem of spherical trigonometry in spherical $\triangle gfh$ as

$$\frac{\sin\omega}{\sin(2\pi - \varphi_4)} = \frac{\sin\left(\frac{\pi-\beta}{2}\right)}{\sin\tau} \tag{7}$$

With Supplementary Equations 2 and 7, the dihedral angle τ is calculated as

$$\tau = \sin^{-1}\left[\frac{\sin\varphi_2\cos(\beta/2)}{\sin\omega}\right] \tag{8}$$

In spherical $\triangle efh$, σ, β and ω are the arc lengths of $\overset{\frown}{eh}$, $\overset{\frown}{ef}$ and $\overset{\frown}{fh}$, respectively. θ presents the dihedral angle between facet oef and facet ofh. Through the Pythagorean theorem, the relationship

between arc length σ and structural height H is expressed as

$$\cos\left(\frac{\sigma}{2}\right) = \frac{2\sin\left(\frac{\beta}{2}\right)\sqrt{\left(l/2\tan\frac{\beta}{2}\right)^2 - \left(\frac{H}{2}\right)^2}}{l} \tag{9}$$

Using Supplementary Equations 3 and 9, the arc length σ is simplified as

$$\sigma = 2\cos^{-1}\frac{2\sin\frac{\beta}{2}\sqrt{\left(\frac{l}{2\tan\frac{\beta}{2}}\right)^2 - \left(\frac{H}{2}\right)^2}}{l} = 2\cos^{-1}\left(\cos\frac{\alpha}{2}\cos\frac{\varphi_2}{2}\right) \tag{10}$$

Inspecting the geometric relationship in Supplementary Fig. 4(b), the dihedral angle θ can be determined with the cosine theorem of spherical trigonometry in spherical $\triangle efh$ as

$$\theta = \cos^{-1}\frac{\cos\sigma - \cos\omega\cos\beta}{\sin\omega\sin\beta} \tag{11}$$

As shown in Supplementary Fig. 4(b), the dihedral angle φ_3 is the difference between the dihedral angle θ and dihedral angle τ. Consequently, the dihedral angle φ_3 can be expressed as a function of the dihedral angle φ_2 and plane geometric variable β:

$$\varphi_3 = \cos^{-1}\frac{\cos\sigma - \cos\omega\cos\beta}{\sin\omega\sin\beta} - \sin^{-1}\frac{\sin\varphi_2\cos(\beta/2)}{\sin\omega} \tag{12}$$

Since φ_2 can be expressed as a function of H, as shown in Supplementary Equation 4, φ_3 is also only determined by the origami height H.

If $2\angle eom = 2\angle fog \leq \angle eof$ $(2\beta \leq \pi)$ in origami, crease om and crease og will not collide until the whole structure reaches a flat foldable state. In other words, the self-locking phenomenon will occur due to the collision between crease om and crease og in the process of axial deformation when $2\angle eom = 2\angle fog > \angle eof$ $(2\beta > \pi)$. In this situation, the self-locking height can be determined as

$$H_{\text{sl}} = \frac{l\sin\vartheta}{\tan(\beta/2)} \tag{13}$$

where ϑ is the dihedral angle between the **xy** plane and plane efo. Moreover it can be expressed as

$$\vartheta = \cos^{-1}[\tan\frac{\beta}{2}] \tag{14}$$

3. Evaluation criterion of approximate synchronous deformation

As described in the main text, the origami is constructed by modifying and fusing Miura-ori and Waterbomb-base elements. Fusion, actually an entire coincidence of facets, makes subcells' folding properties couple with each other. If all facets of origami do not deform during folding, the structural folding belongs to rigid folding. Ideally, structural folding belongs to nonrigid folding as long as one facet deforms. For simplification, the nonrigid folding can still be approximated as rigid folding if the deformation of facets is small. To establish boundary conditions for the rigid folding analytical model proposed in the above section, the evaluation criterion for approximate rigid folding is investigated in this section.

The origami folds as a single degree of freedom in the **z**-direction. At any structural height, the origami is not only rotationally symmetrical along the **z**-axis but also vertically symmetrical to the plane of height $H/2$. As such, the analysis can be simplified and localized between symmetry planes, as shown in Supplementary Fig. 5 and Supplementary Fig. 6. There are two types of rotational symmetry planes for the origami element. The first passes through the top and bottom vertex of the modified Miura-ori cell and the central axis of the origami element, that is, datum 1 as shown in Supplementary Fig. 5(a). The second passes through the central vertex of the modified Waterbomb-base element and the central axis of the origami element (i.e., datum 2 plotted in Supplementary Fig. 5(a)). In the process of folding, facets iii and iv are always perpendicular to the first class of rotational symmetry plane datum 1. Facets v and vi are always perpendicular to datum 2. In the fourth quadrant of the coordinate system, the dihedral angle between adjacent rotational symmetry planes datum 1 and datum 2 is always kept the same as π/n. In addition, the same type of vertex located at the same height passes through the same circle, whose center is on the structural centre axis. For example, vertex v_0 passes through circle C1, as shown in Supplementary Fig. 5(b). Similarly, vertex v_8 and vertex v_9 locate at the same circle C1, as shown in Supplementary Fig. 6(b).

To evaluate structural approximate rigid folding for the origami element, the entire coincidence of facets i and ii, as shown in Supplementary Fig. 2(a), is relaxed to the coincidence of two vertices at both ends of the adjacent subcell. The coincidence of vertices means that vertex v_0 coincides with vertex v_8 and vertex v_4 coincides with vertex v_{10} after decomposition, as shown in Supplementary Figs. 5 and 6. We define sharing crease 4 in modified Miura-ori as crease $4'$ and sharing crease 4 in modified Waterbomb-base as crease $4''$. If the folding of origami belongs to rigid folding, crease $4'$ in the modified Miura-ori cell and crease $4''$ in the Waterbomb-base element always move synchronously and coincide

during folding. The modified Miura-ori and Waterbomb-base elements realize fully synchronous deformation. If the folding of origami belongs to approximate rigid folding, there will be a small deviation between crease $4'$ and crease $4''$ and the modified Miura-ori and Waterbomb-base elements deform with a approximate synchronous mode. Therefore, the position of crease $4'$ and crease $4''$ becomes the most representative parameter to evaluate the approximate synchronous deformation property for the origami element.

A. The position of crease $4'$ and crease $4''$

Crease $4'$ is determined by vertex v_2 located in circle C2 and vertex v_3 on circle C3 in the modified Miura-ori cell, as shown in Supplementary Fig. 5(b). The centroid of the bottom surface in the origami element is selected as the coordinate origin. The positive direction of the coordinates is shown in Supplementary Fig. 5(a). Since vertex v_2 and vertex v_3 are always on the symmetry plane in the **z**-direction during folding, their coordinates on the **z**-axis are always equal to $H/2$. Before calculating the coordinates of vertex v_2 and vertex v_3 on the **xy** plane, vertex v_0 is chosen as the calculated reference point, and its coordinate is determined to be $\left[\frac{l}{2\sin(\pi/n)}, -\frac{\pi}{n}, H\right]$.

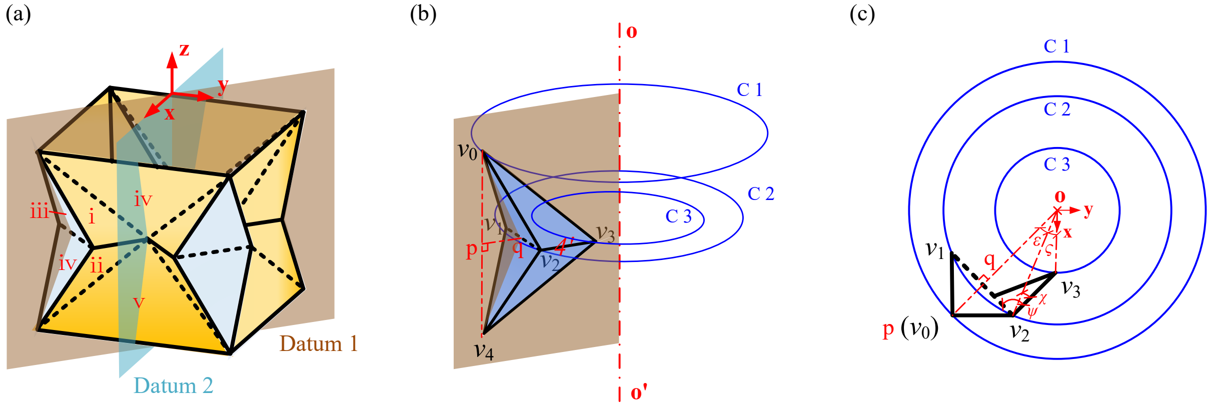

Supplementary Fig. 5: **The position schematic for vertex of modified miura-ori in the origami element**. (a) Schematic diagram of symmetrical rotational planes and coordinate system's positive direction for the origami with a height of H. (b) The modified Miura-ori cell in the coordinate system for position determination of crease $4'$. The red stippled line indicates the central axis of origami. The red dotted line is the auxiliary line. The blue circles indicate the radial positions of different vertices in the structure. (c) Top view of modified Miura-ori elements in origami. Similarly, the red dotted line is the auxiliary line.

First, we determine the coordinate of v_2. According to the Pythagorean theorem, the length of line

pq is calculated as

$$l_{\mathrm{pq}} = l_1 \sin\alpha \cos\left(\frac{\varphi_2}{2}\right) \tag{15}$$

Under vertex coincidence, the height of modified cells only has a certain correspondence with the dihedral angle of the creases in the respective cell. Therefore, the relationship between φ_2 and H is determined by the sines law and expressed as follows:

$$\varphi_2 = 2\sin^{-1}\left[\frac{H}{2l_1 \sin\alpha}\right] \tag{16}$$

Similarly, the radius r_{C2} of circle C2 is calculated using Pythagorean theorem, and the angle ε between line $\mathbf{o}v_0$ and line $\mathbf{o}v_2$ is calculated through the trigonometric function method as

$$\begin{cases} r_{\mathrm{C2}} = \sqrt{l_{\mathbf{o}\mathrm{q}}^2 + l_{\mathrm{q}v_1}^2} \\ \varepsilon = \tan^{-1}\left(l_{\mathrm{q}v_1}/l_{\mathbf{o}\mathrm{q}}\right) \end{cases} \tag{17}$$

where the length of line $\mathbf{o}$q is calculated by $l_{\mathbf{o}\mathrm{q}} = l/2\sin(\pi/n) - l_{\mathrm{pq}}$, and the length of line qv_2 is solved as $l_{\mathrm{q}v_2} = l_2/2$. Inspecting Supplementary Figs. 5(b) and 5(c), the coordinate of vertex v_2 is then determined as $[r_{\mathrm{C2}}, \varepsilon - \pi/n, H/2]$.

Next, we determine the coordinates of vertex v_3 during deformation. Based on the edge cosine formula of spherical trigonometry, the relationship among angle ψ between line v_1v_2 and line v_2v_3, dihedral angle φ_1 and dihedral angle φ_2 in spherical $\triangle v_0v_1v_3$ is given by

$$\cos(2\pi - \varphi_1) = -\cos\left(\pi - \frac{\varphi_2}{2}\right)\cos\left(\frac{\varphi_2}{2}\right) + \sin\left(\pi - \frac{\varphi_2}{2}\right)\sin\left(\frac{\varphi_2}{2}\right)\cos\psi \tag{18}$$

Then, the angle ψ is calculated as

$$\psi = \cos^{-1}\left[\frac{\cos\varphi_1 - \cos^2(\varphi_2/2)}{\sin^2(\varphi_2/2)}\right] \tag{19}$$

where the dihedral angle φ_1 can be expressed as a function of the dihedral angle φ_2 (Supplementary Equation 2). The angle χ between line $\mathbf{o}v_2$ and line $\mathbf{o}v_3$ is given by $\psi + \varepsilon - \pi/2$. Through the relationship among the side lengths and angles of $\triangle \mathbf{o}v_2v_3$, the radius of circle C3 and angle ς between line $\mathbf{o}v_2$ and

line $\mathbf{o}v_3$ are, respectively, expressed as

$$\begin{cases} r_{\mathrm{C3}} = \sqrt{l_{\mathbf{o}v_2}^2 + l_4^2 - 2l_{\mathbf{o}v_2} l_4 \cos\chi} \\ \varsigma = \sin^{-1}\left(\dfrac{l_4 \sin\chi}{r_{\mathrm{C3}}}\right) \end{cases} \tag{20}$$

Thus, the coordinate of vertex v_3 can be expressed as $[r_{\mathrm{C3}}, \varepsilon + \varsigma - \pi/n, H/2]$.

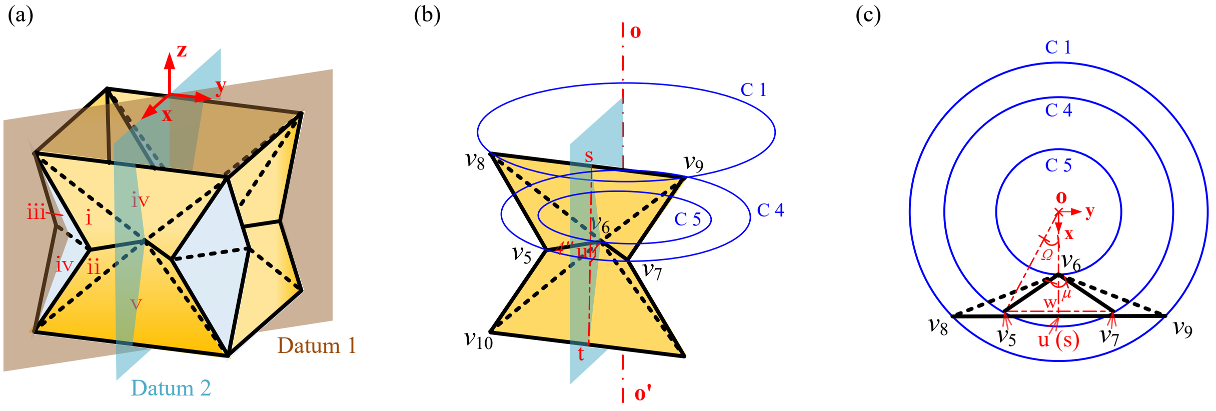

Supplementary Fig. 6: **The position schematic of vertex for modified waterbomb-base in the origami element**. (a) Schematic diagram of symmetrical rotational planes and coordinate system's positive direction for the origami with a height of H. (b) The modified Waterbomb-base element in the coordinate system for position determination of crease $4''$. The red stippled line indicates the central axis of origami. The red dotted line is the auxiliary line. The blue circles indicate the radial positions of different vertices in the structure. (c) Top view of modified Waterbomb-base elements in origami. Similarly, the red dotted line is the auxiliary line.

Crease $4''$ is determined by vertex v_5 on circle C4 and vertex v_6 on circle C5 in the modified Waterbomb-base element, as shown in Supplementary Fig. 6(b). Since vertices v_5 and v_6 are on the symmetry plane in the **z**-direction during folding, their coordinates on the **z**-axis are $H/2$. Before calculating the coordinates of vertex v_5 and vertex v_6 in the **xy** plane, vertex s is chosen as the calculated reference point, and its coordinate is $\left[\frac{l}{2\tan(\pi/n)}, 0, H\right]$. The length of line uv_6 is determined with the Pythagoram theorem as

$$l_{\mathrm{u}v_6} = \frac{1}{2}\sqrt{\frac{l^2}{\tan^2(\beta/2)} - H^2} \tag{21}$$

The radius r_{C5} of circle C5 is given by $l/[2\tan(\pi/n)] - l_{\mathrm{u}v_6}$, and the coordinate of vertex v_6 is expressed as $[r_{\mathrm{C5}}, 0, H/2]$.

Next, we determine the coordinate of v_5. To calculate the angle μ through spherical trigonometry, we assume that κ is the dihedral angle between facet $v_5v_6v_7$ and facet $v_6v_7v_8$. η is the dihedral angle between facet $v_6v_7v_9$ and facet $v_6v_7v_8$. ρ is the dihedral angle between facet $v_5v_6v_8$ and facet $v_6v_7v_8$. ζ is the dihedral angle between facet $v_6v_8v_9$ and facet $v_6v_7v_8$. $\varGamma$ is the angle between line v_6v_8 and line v_6v_7. Among them, κ and η have the relationship of $\kappa + \eta = \varphi_4/2$. ρ and ζ have a relationship of $\rho + \zeta = \varphi_3$. Moreover, $\varGamma$, η and ζ are solved through spherical trigonometry as follows:

$$\begin{cases} \varGamma = \cos^{-1}\left[\cos\beta\sin\dfrac{\beta}{2} + \sin\beta\cos\dfrac{\beta}{2}\cos\varphi_3\right] \\ \zeta = \cos^{-1}\left[\dfrac{\sin(\beta/2) - \cos\varGamma\cos\beta}{\sin\varGamma\sin\beta}\right] \\ \eta = \cos^{-1}\left[\dfrac{\cos\beta - \cos\varGamma\sin(\beta/2)}{\sin\varGamma\cos(\beta/2)}\right] \end{cases} \tag{22}$$

where the dihedral angle φ_3 can be expressed as a function of the dihedral angle φ_4 and cell height H based on Supplementary Equations 5, 7, 10 and 12. The dihedral angle φ_4 and structural height H have the relationship

$$\varphi_4 = 2\left[\pi - \sin^{-1}\left(\frac{H}{2h}\right)\right] \tag{23}$$

in a modified Waterbomb-base element. h is the height of $\triangle v_5v_6v_8$ with v_5v_6 as the base and given by the Helen formula

$$h = \frac{2\sqrt{P(P-l_1)(P-l_3)(P-l_4)}}{l_4} \tag{24}$$

where the parameter P is calculated by $P = (l_1 + l_3 + l_4)/2$. Finally, the angle μ between line v_5v_6 and line v_6v_7 is calculated as

$$\mu = \cos^{-1}\left[\frac{\cos\rho + \cos(\varphi_4/2)\cos\kappa}{\sin(\varphi_4/2)\sin\kappa}\right] \tag{25}$$

Finally, the radius r_{C4} of circle C4 and the angle $\varOmega$ between line $\mathbf{o}v_5$ and line $\mathbf{o}v_6$ are determined to be

$$\begin{cases} r_{C4} = \sqrt{l_4^2\sin^2(\mu/2) + l_{\mathbf{o}w}^2} \\ \varOmega = \tan^{-1}\left[\dfrac{l_4\sin(\mu/2)}{l_{\mathbf{o}w}}\right] \\ l_{\mathbf{o}w} = \dfrac{l}{2\tan(\pi/n)} - l_{uv_6} + l_4\cos(\mu/2) \end{cases} \tag{26}$$

The coordinate of v_5 can be expressed as $[r_{C4}, -\varOmega, H/2]$.

B. The analysis of approximate synchronous deformation

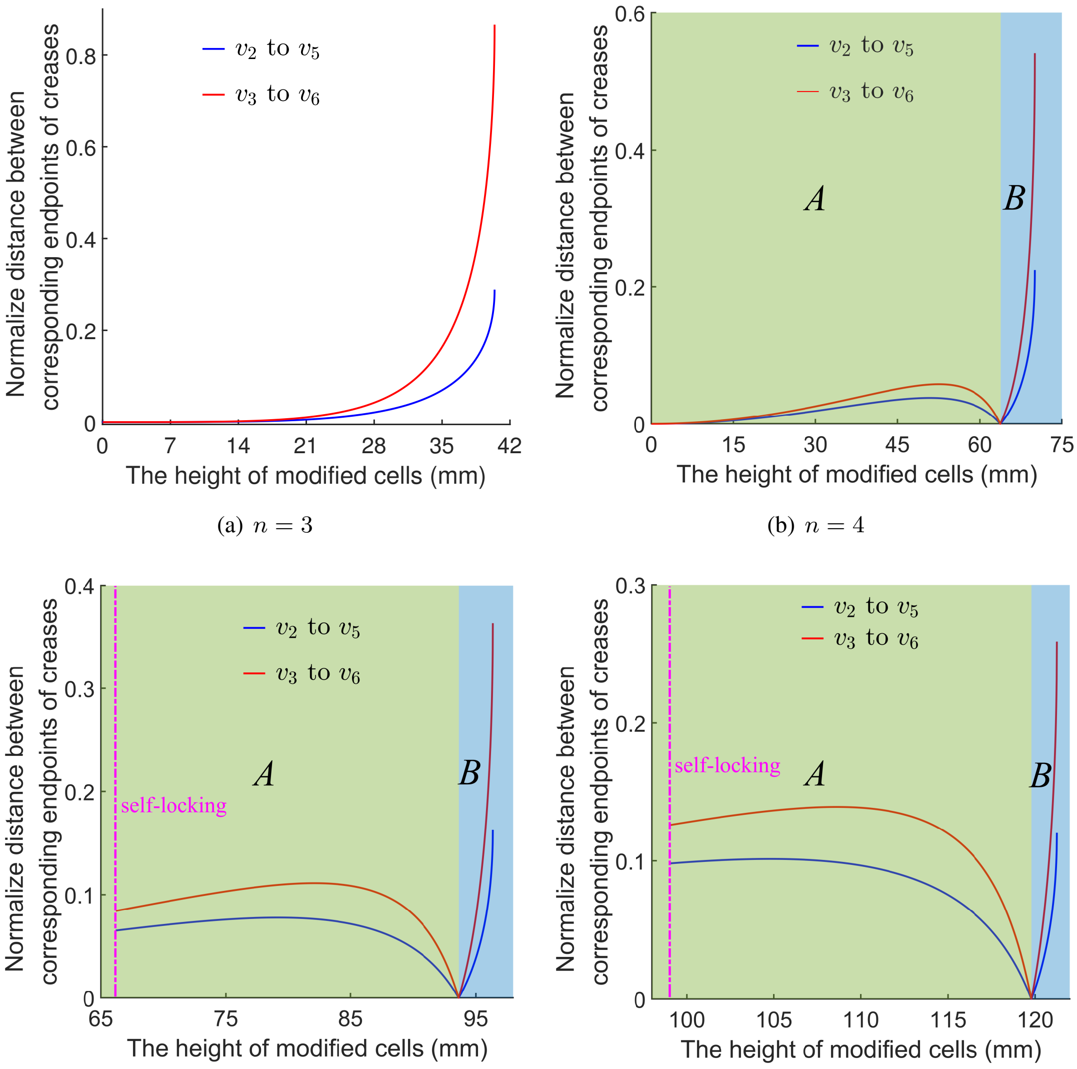

(a) $n = 3$ (b) $n = 4$

(c) $n = 5$ (d) $n = 6$

Supplementary Fig. 7: **The normalized distance between the corresponding endpoints of crease** $4'$ **and crease** $4''$ **on the xy plane at the height of** $H/2$ **for the origami element with (a)** n **= 3, (b)** n **= 4, (c)** n **= 5 and (d)** n **= 6 under vertex coincidence at both ends of the adjacent subcell**.

The normalized distance between corresponding endpoints of creases (v_2 and v_5, v_3 and v_6) is introduced to measure the distance between crease $4'$ and crease $4''$, which ignores the influence of external structural dimensions and is defined as the ratio of the distance between the corresponding endpoints to the radius of the circumscribed circle for the uppermost cross section of the origami element. For

origami with n = 3, 4, 5 and 6 under vertex coincidence at both ends of adjacent subcells, the normalized distances between corresponding endpoints varied by the structural height are plotted in Supplementary Fig. 7. In addition, the positions of crease $4'$ and crease $4''$ on the **xy** plane at the height of $H/2$ for origami with n = 3, 4, 5 and 6 under vertices coinciding at both ends of the adjacent subcell are obtained and presented in Supplementary Figs. 8, 9, 10 and 11, respectively. The normalized distance from v_2 to v_5 and the normalized distance from v_3 to v_6 maintain a consistent variation trend. For the structure with n = 3 under vertices coinciding at both ends of the adjacent subcell, the normalized distance is 0 when the subcell height H is equal to 0 mm. In other words, crease $4'$ in the modified Miura-ori cell and crease $4''$ in the modified Waterbomb-base element coincide, as shown in Supplementary Fig. 8(a). With the increase in structural height H, the normalized distance between the corresponding endpoints of crease $4'$ and crease $4''$ continues to increase until the subcell height reaches its maximum value. Moreover, the increasing rate increases drastically at larger structural heights. Thus, the distance between crease $4'$ and crease $4''$ keeps increasing as shown in Supplementary Fig. 8(b), (c) and (d). For the structure with n $\geq$ 4 under vertices coinciding at both ends of the adjacent subcell, the normalized distance difference between the corresponding endpoints of crease $4'$ and crease $4''$ is slightly different from origami with n = 3. The normalized distance first increases to a small extremum, then decreases to zero, and finally increases sharply to a large extremum until the structural height H reaches its maximum value as shown in Supplementary Figs. 9, 10 and 11. Crease $4'$ and crease $4''$ coincide twice during folding for the structure with n = 4 under vertex coincidence at both ends of the adjacent subcell.

The distance between v_2 and v_5 is always greater than that between v_3 and v_6 when there is a deviation between crease $4'$ and crease $4''$. In other words, crease $4'$ and crease $4''$ are not always parallel and vary by cell height. In this case, stretching, bending and torsion occur simultaneously for the facets of the origami element after fusion to realise structural deformation coordination of the folding characteristics between modified subcells. It can be estimated based on the distance between the corresponding endpoints of crease $4'$ and crease $4''$ that facet deformation is strongly dependent on the cell height. The structure only achieves undamaged deformation under the allowable crease distance of the structural material. Otherwise, the structure is damaged due to excessive facet deformation during folding. For the origami element with n = 3, the facets' deformation at a small and medium structural height is much smaller than that at a large structural height. For the origami element with $n \geq 4$, the deformation height can be divided into two regions A and B with the deformation height under zero facet deformation as the demarcation node to distinguish the difference in facet deformation. The normalized distance betweeen

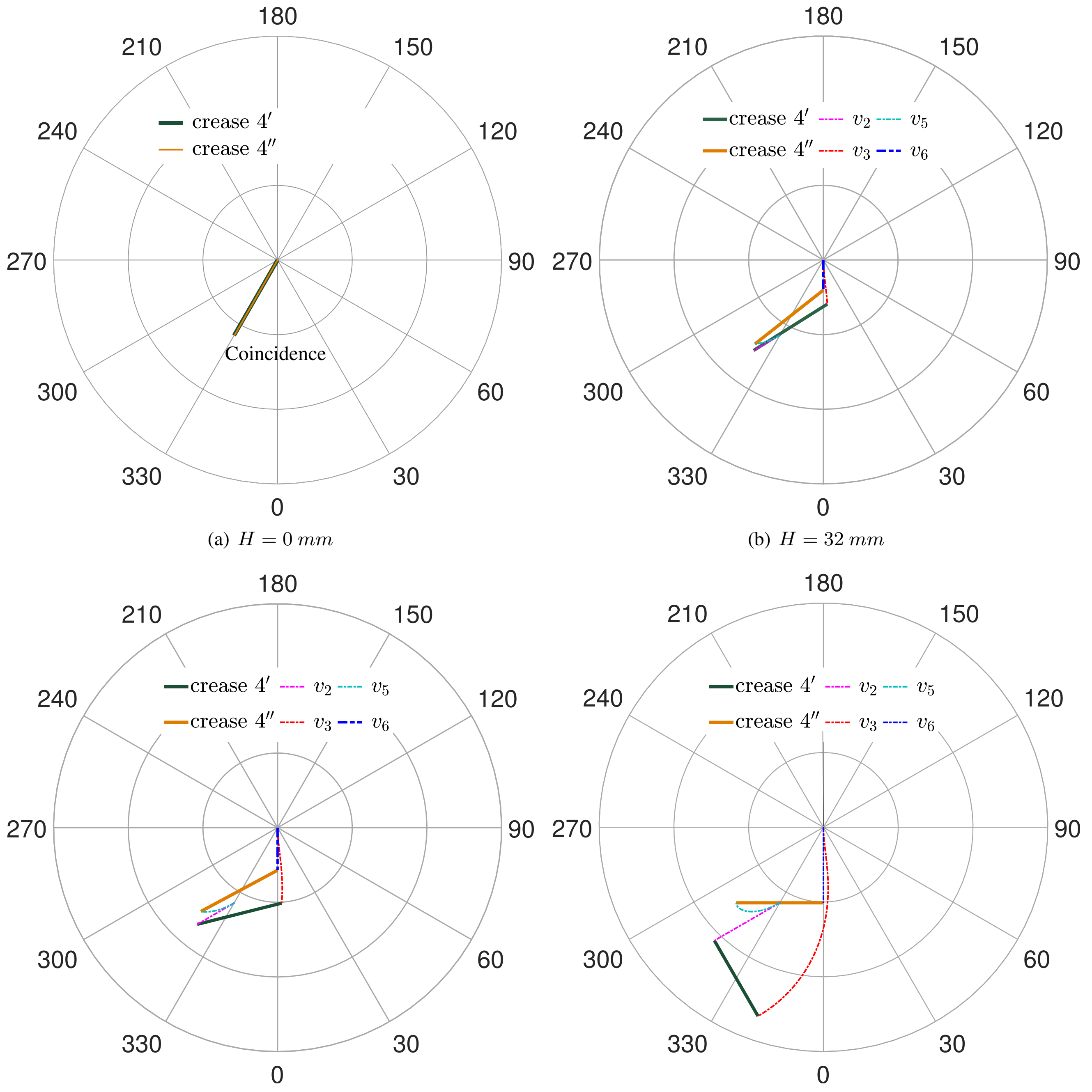

(a) $H = 0\,mm$ (b) $H = 32\,mm$

(c) $H = 36\,mm$ (d) $H = 40\,mm$

Supplementary Fig. 8: **The position of crease** $4'$ **and crease** $4''$ **at the xy plane at the height of** $H/2$ **for origami element with** n **= 3 under vertices coinciding at both ends of the adjacent subcell when their height** H **= (a) 0 mm, (b) 32 mm, (c) 36 mm and (d) 40 mm.** The solid lines indicate crease $4'$ and crease $4''$. The dashed lines represent the trajectory of the endpoints of crease $4'$ and crease $4''$.

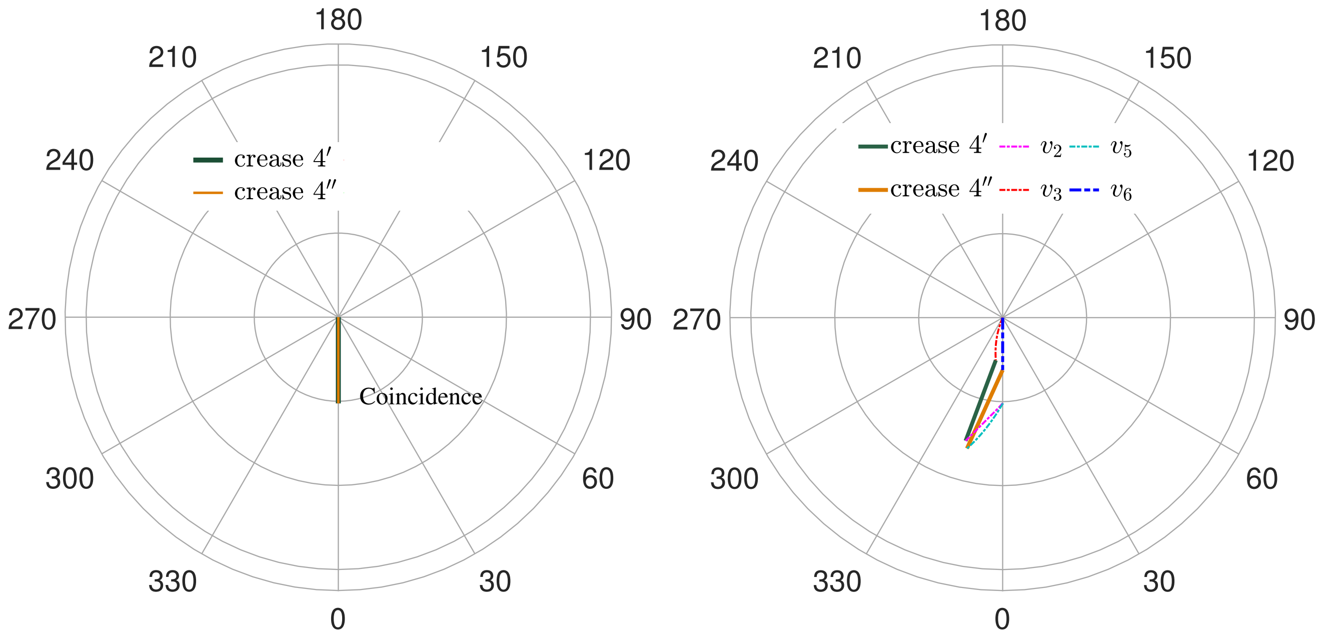

(a) $H = 0\,mm$

(b) $H = 53.6\,mm$

(c) $H = 64\,mm$

(d) $H = 70\,mm$

Supplementary Fig. 9: **The position of crease** $4'$ **and crease** $4''$ **at the xy plane at the height of** $H/2$ **for origami element with** n **= 4 under vertices coinciding at both ends of the adjacent subcell when their height** H **= (a) 0 mm, (b) 53.6 mm, (c) 64 mm and (d) 70 mm.**. The solid lines indicate crease $4'$ and crease $4''$. The dashed lines represent the trajectory of the endpoints of crease $4'$ and crease $4''$.

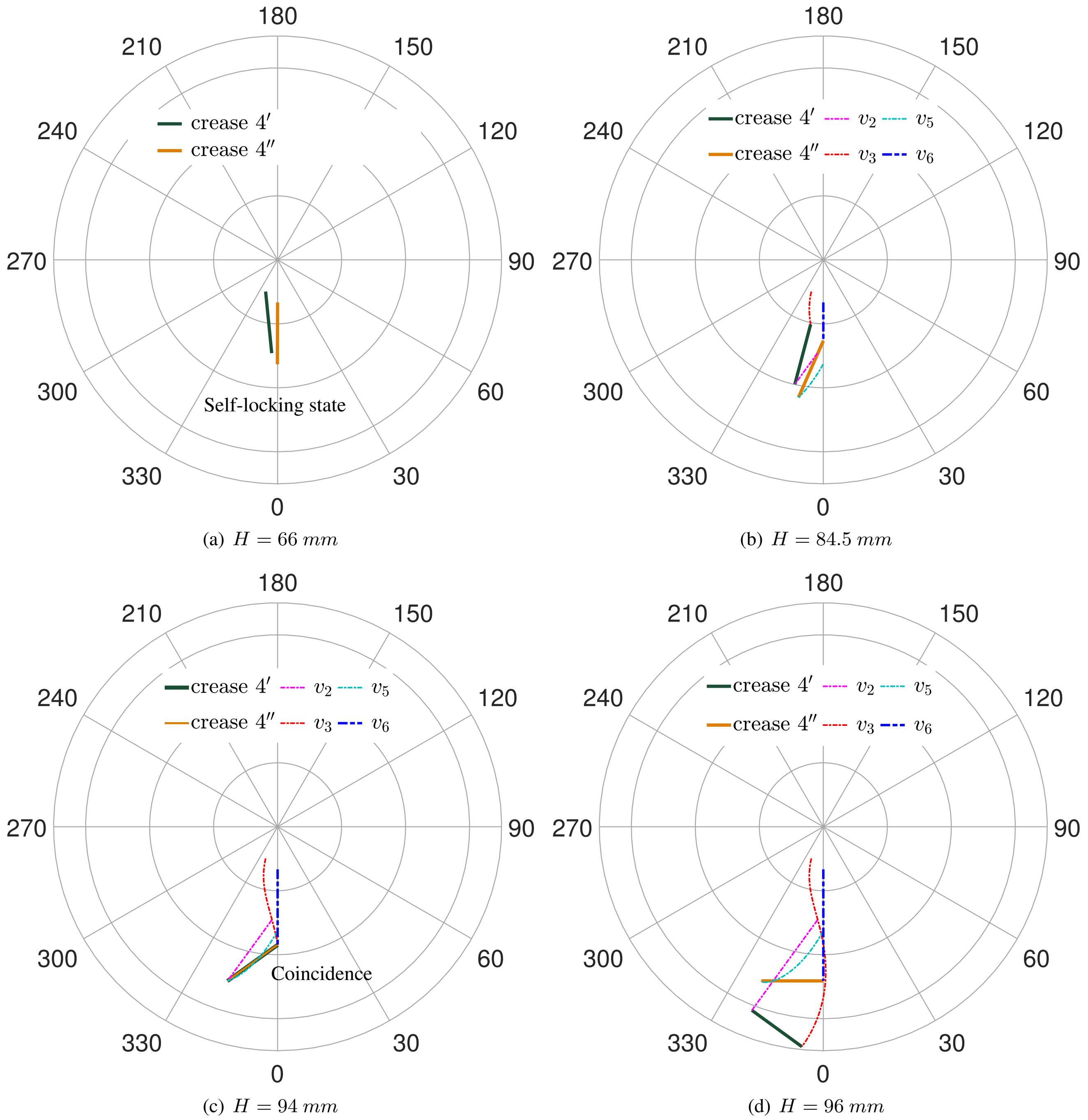

(a) $H = 66\,mm$ (b) $H = 84.5\,mm$

(c) $H = 94\,mm$ (d) $H = 96\,mm$

Supplementary Fig. 10: **The position of crease** $4'$ **and crease** $4''$ **at the xy plane at the height of** $H/2$ **for origami element with** n **= 5 under vertices coinciding at both ends of the adjacent subcell when their height** H **= (a) 66 mm, (b) 84.5 mm, (c) 94 mm and (d) 96 mm.** The solid lines indicate crease $4'$ and crease $4''$. The dashed lines represent the trajectory of the endpoints of crease $4'$ and crease $4''$.

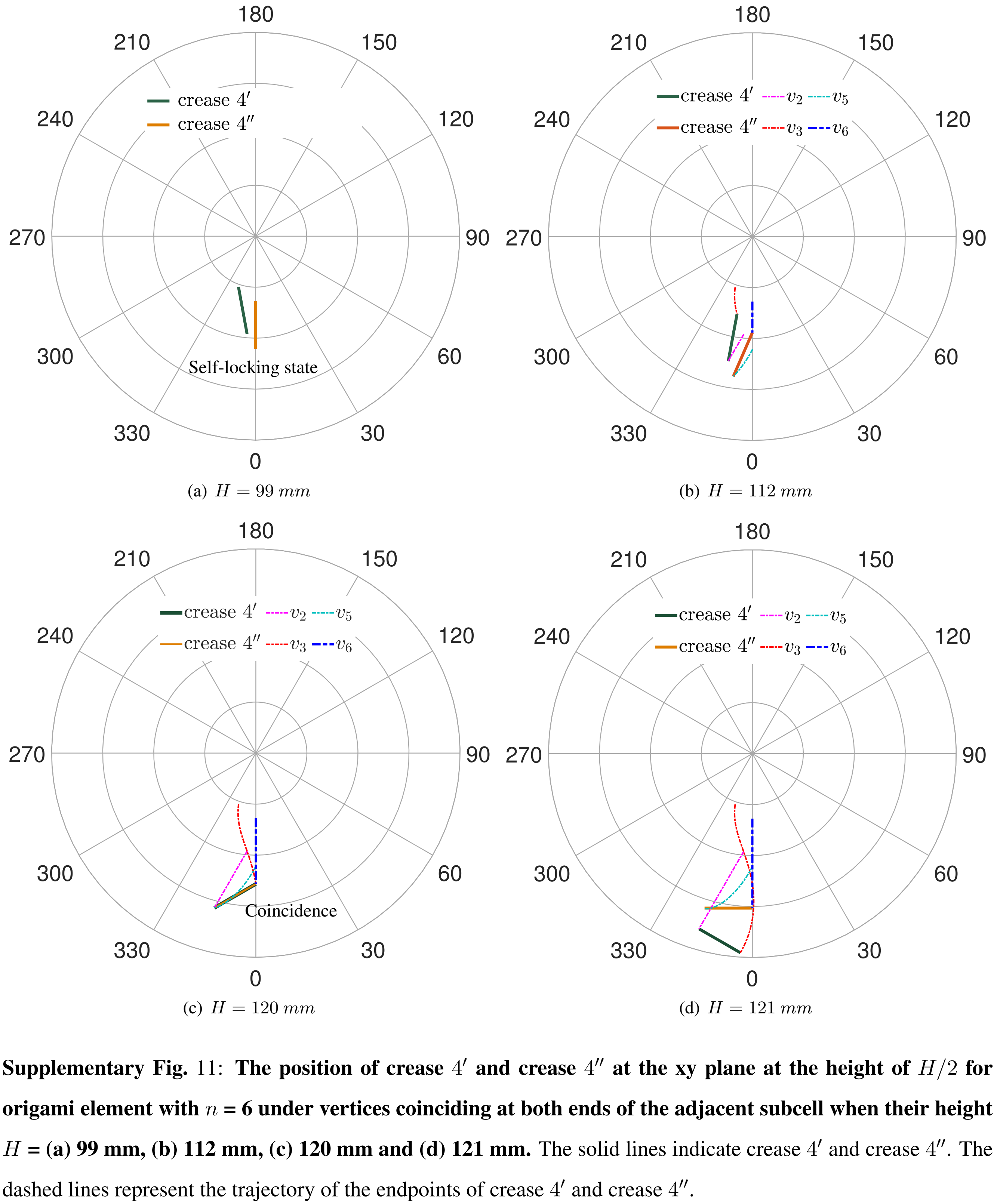

(a) $H = 99\ mm$ (b) $H = 112\ mm$ (c) $H = 120\ mm$ (d) $H = 121\ mm$

Supplementary Fig. 11: **The position of crease** $4'$ **and crease** $4''$ **at the xy plane at the height of** $H/2$ **for origami element with** n **= 6 under vertices coinciding at both ends of the adjacent subcell when their height** H **= (a) 99 mm, (b) 112 mm, (c) 120 mm and (d) 121 mm.** The solid lines indicate crease $4'$ and crease $4''$. The dashed lines represent the trajectory of the endpoints of crease $4'$ and crease $4''$.

corresponding endpoints for the origami element with n = 4, 5, 6, 7, 8, 9 and 10 under vertex coincidence

Supplementary Table 1: **Structural deformation measured by vertex distance**.

n	region A		region B	
	Normalized distance between v_2 and v_5	Normalized distance between v_3 and v_6	Normalized distance between v_2 and v_5	Normalized distance between v_3 and v_6
4	0.0377	0.0580	0.2242	0.5412
5	0.0767	0.1111	0.1625	0.3633
6	0.0992	0.1390	0.1201	0.2588
7	0.1109	0.1516	0.0915	0.1931
8	0.1160	0.1558	0.0717	0.1493
9	0.1172	0.1554	0.0575	0.1188
10	0.1195	0.1519	0.0471	0.0967

at both ends of adjacent subcells in regions A and B are given in Supplementary Table 1 when the normalized creases $4'$ and crease $4''$ are largest. The data indicate that the origami element with n = 4, 5, 6 and 7 may exhibit larger facets' deformation in region B. However, the origami element with $n \geq 8$ will undergo larger facets' deformation in interval A. Moreover, the facet deformation is relatively small for the origami element with a larger number side n. We choose the allowable normalized distance of 0.16, which is equivalent to 8% of the radial dimension of the origami element, to ensure the foldability of the origami with any n. The deformable height in interval B for the origami element with $n \geq 4$ is less than 4 mm and can be neglected. Thus, the maximum height $H_{\max}$ of origami with n = 3, 4, 5, 6, 7, 8, 9 and 10 reaches 35 mm, 64 mm, 94 mm, 120 mm, 145 mm, 168 mm, 192 mm and 215 mm, respectively, for the same side length l = 70 mm.

4. Potential energy and reaction force

Longitudinal deformation appears if the origami model bears axial force. It is assumed that the energy accumulation or release of the origami element mainly occurs at the creases. Each crease is considered as a compliant small-length flexural pivot. With these two assumptions, the variation in potential energy with dihedral angles of the creases follows the Hookean energy expression [2]. Thus, the potential energy for the origami element can be expressed as

$$E_{\text{tot}} = \frac{n}{2}\sum_{j=1}^{4} m_j k_j (\varphi_j - \overline{\varphi}_j)^2 \tag{27}$$

where n is the side number of interweaved girdling, $\overline{\varphi}_j$ is the spatial initial dihedral angle corresponding to crease j (j = 1, 2, 3, 4), and m_j is the number of creases j in an element. k_j is the rotational stiffness of crease j and is given by $El_j h_j^3/12b_j$. E is Young's modulus of crease material. l_j, h_j and b_j represent the length, thickness and width of crease j, respectively.

In addition to the external axial force, only the internal force does work along the corresponding virtual displacement during deformation. Therefore, the external axial reaction force, acting on the top or bottom sides, can be obtained by the principle of virtual work [3] as follows:

$$F_{\text{a}} = -n\sum_{j=1}^{4} m_j k_j (\varphi_j - \overline{\varphi}_j)\frac{d\varphi_j}{dH} \tag{28}$$

where the kinematic coefficients $d\varphi_j/dH$ are calculated from Supplementary Equations 2, 4 and 12 as

$$\frac{d\varphi_1}{dH} = -\frac{2\cos\alpha}{(1+\cos\xi)}\frac{d\varphi_2}{dH} \tag{29}$$

$$\frac{d\varphi_2}{dH} = \frac{2\tan(\beta/2)}{\sqrt{l^2 - [H\tan(\beta/2)]^2}} \tag{30}$$

$$\frac{d\varphi_3}{dH} = -\left(\frac{1}{\sqrt{1-g^2}}\frac{dg}{dH} + \frac{1}{\sqrt{1-r^2}}\frac{dr}{dH}\right) \tag{31}$$

$$\frac{d\varphi_4}{dH} = -\frac{d\varphi_2}{dH} \tag{32}$$

where the expressions of intermediate variables g and r and their derivatives are presented in the following discussion.

Since the complex expression of φ_3 is shown in Supplementary Equation 12, two variables g and r

are adopted to simplify the derivation of the reaction force. In the process of calculating the reaction force, let $\varphi_3 = \cos^{-1}g - \sin^{-1}r$. Therefore, g and r are given by

$$g = \frac{\cos\beta - 2(H/l)^2\sin^2(\beta/2) - \cos\omega\cos\beta}{\sin\omega\sin\beta} \tag{33}$$

$$r = \frac{\sin\varphi_2\cos(\beta/2)}{\sin\omega} \tag{34}$$

For the expression of $d\varphi_3/dH$ as shown in Supplementary Equation 31, the coefficients dg/dH and dr/dH are determined from Supplementary Equations 33 and 34 as

$$\frac{dg}{dH} = \frac{\cos\beta}{\sin\beta}\frac{d\omega}{dH} - \frac{4H\sin^2(\beta/2)}{l^2\sin\omega\sin\beta} - \frac{\left(\cos\beta - 2(H/l)^2\sin^2(\beta/2) - \cos\omega\cos\beta\right)\cos\omega}{\sin^2\omega\sin\beta}\frac{d\omega}{dH} \tag{35}$$

$$\frac{dr}{dH} = \cos(\beta/2)\left(\frac{d\varphi_2}{dH}\frac{\cos\varphi_2}{\sqrt{1-Q^2}} - \frac{d\omega}{dH}\frac{\sin\varphi_2\cos\omega}{1-Q^2}\right) \tag{36}$$

$$\frac{d\omega}{dH} = \frac{1}{\sqrt{1-Q^2}}\frac{d\varphi_2}{dH}\sin\varphi_2\cos^2\frac{\beta}{2} \tag{37}$$

where the intermediate variable $Q = \sin^2(\beta/2) + \cos^2(\beta/2)\cos\varphi_2$.

5. Crease mechanical contribution

Based on the approximate synchronous deformation, only the deformation at the crease produces a reaction force to resist external forces inducing the folding of origami element. Moreover, the mechanical responses of different creases are not the same based on their unique folding characteristics. From Supplementary Equation 28, the reaction force of the whole origami element is superimposed by that produced by each type of crease. Therefore, we separately investigate the mechanical component ΣF_j of all crease deformation on the reaction force of origami element, as shown in Supplementary Fig. 12.

To investigate the influence of the initial dihedral angle for each type of crease j on the structural mechanical response, the contribution factor of each crease j is introduced and defined as the product of the crease number, length and change rate of the dihedral angle by the height at the beginning folding. The contribution factor can reflect the weight coefficient of the reaction force calculation formula for the certain crease j. From the main text, the origami element exhibits two types of mechanical responses

Supplementary Table 2: **The contribution factor of each Crease** j **for origami element with** n **= 4 at the beginning folding**

Properties	Crease 1	Crease 2	Crease 3	Crease 4
l ($\times 10^{-3}$ m)	37.8837	28.9948	49.4975	20.5025
num	16	4	16	8
$\frac{d\varphi_j}{dH}$ (rad/m)	90.4222	69.0918	83.4415	69.0918
contribution factor (rad)	54.8084	8.0132	66.0823	11.3324

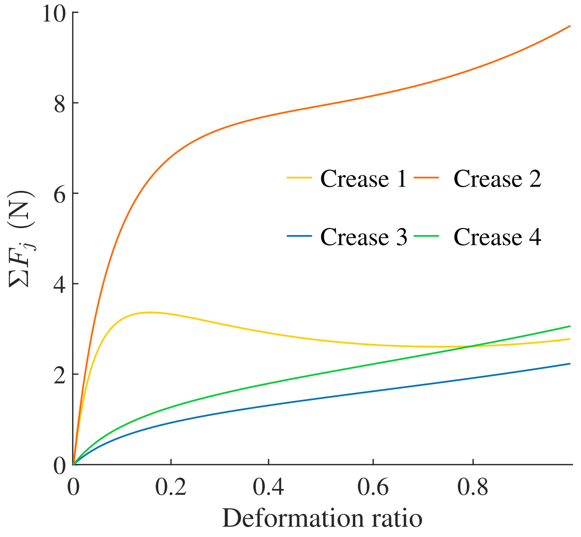

Supplementary Fig. 12: **The reaction force produced by crease** j **varied by the deformation ratio for the origami element with** n **= 4.**

— rigid-elastic response and complete-elastic response. If the reaction force of the origami element is greater than zero when the structure begins to deform, its mechanical response belongs to the rigid-elastic response. Otherwise, the mechanical response of origami element belongs to a complete-elastic response. The initial value of the reaction force has a great impact on the type of structural mechanical response. Therefore, the contribution factor of crease j at the initial folding reflects the influence of its initial dihedral angle variation on the mechanical response. The contributing factors of all creases for the origami element with n = 4 are presented in Supplementary Table 2. It is obvious that the selection of the initial dihedral angle of crease 1 and crease 3 has a greater impact on the structural mechanical response. By comparison, the selection of the initial dihedral angle of crease 2 and crease 4 has a weak

(a)

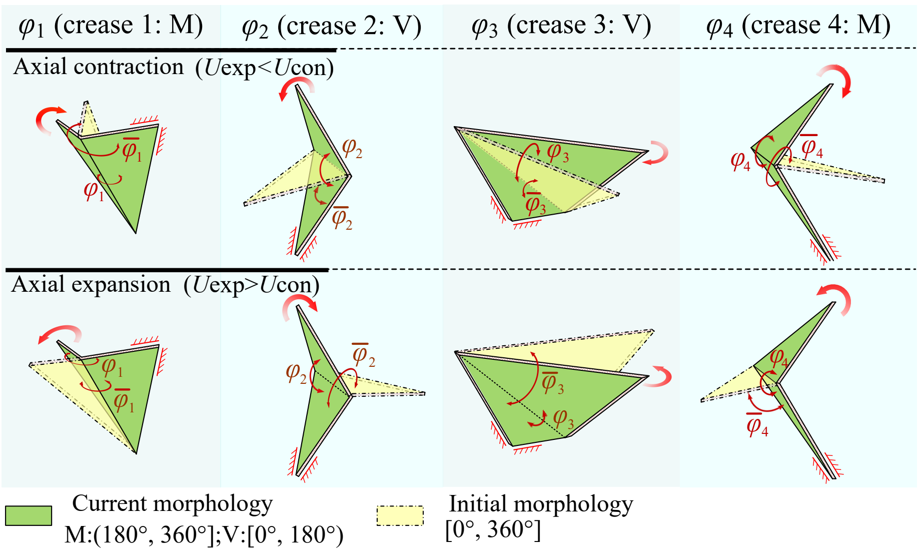

(b)

Origami element	Potential energy	H_{stable}	$\overline{\varphi}_1$	$\overline{\varphi}_2$	$\overline{\varphi}_3$	$\overline{\varphi}_4$
C_I	$U_{\text{exp}}=U_{\text{con}}$	*0*	360º	0º	0º	360º
C_{II}		$0.6H_{\text{max}}$	170º	0º	0º	360º
C_{III}		H_{max}	130º	0º	0º	180º
R	$U_{\text{exp}}>U_{\text{con}}$	H_{max}	0º	0º	0º	0º

Supplementary Fig. 13: **(a) contraction and expansion trends of different creases with different initial dihedral angle and (b) their effects on mechanical response of origami element.**

influence on the structural mechanical response.

For the origami element with n = 4, the reaction force produced by crease 3 or crease 4 is relatively small and increases slowly with increasing deformation ratio. The reaction force produced by crease 1 or crease 2 increases rapidly during the initial deformation and reaches a large value. The initial reaction force produced by crease 2 is more than twice that produced by crease 1. As the deformation ratio increases, the increasing rate of the reaction force produced by crease 2 becomes weakened. The reaction force produced by crease 1 begins to decrease and then becomes relatively stable. Overall, the force produced by crease 2 contributes the most to the reaction force of origami, followed by crease 1 and crease 4, and the smallest one is crease 3. When the deformation ratio is small, the rapidly rising forces of

crease 1 and crease 2 introduce the large stiffness of the origami element. At a larger deformation ratio, even though the reaction force of crease 1 is reduced, the increasing reaction forces of other creases still lead to an increase in the reaction force for the origami element.

6. Programmable stable state and mechanical response

From the main text, the mechanical property of the origami element is a superimposed result of the contraction and expansion effect of different creases caused by the difference between the initial morphology and current morphology. The initial dihedral angles of $\bar{\varphi}_1$, $\bar{\varphi}_2$, $\bar{\varphi}_3$ and $\bar{\varphi}_4$ are considered important parameters to achieve the mechanical property programming of the origami element with fixed side number n.

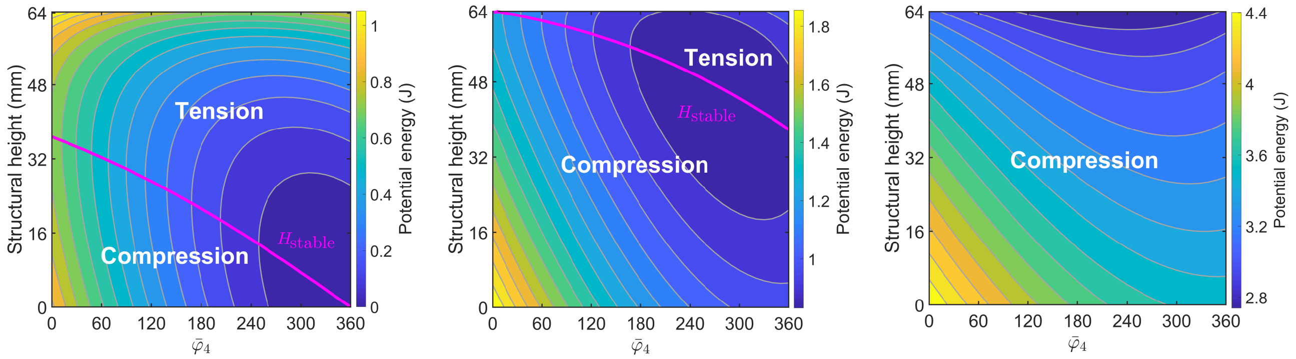

(a) $\bar{\varphi}_1 = 360°$ ($U_{\text{exp}} = U_{\text{con}}$ at H_{stable}) (b) $\bar{\varphi}_1 = 170°$ ($U_{\text{exp}} = U_{\text{con}}$ at H_{stable}) (c) $\bar{\varphi}_1 = 0°$ ($U_{\text{exp}} > U_{\text{con}}$ at H_{max})

Supplementary Fig. 14: **Contour plot of potential energy varied with $\bar{\varphi}_4$ under (a) $\bar{\varphi}_1$ = 360 ° and $\bar{\varphi}_2$ = $\bar{\varphi}_3$ = 0 °, (b) $\bar{\varphi}_1$ = 170 ° and $\bar{\varphi}_2$ = $\bar{\varphi}_3$ = 0 °, (c) $\bar{\varphi}_1$ = 0 ° and $\bar{\varphi}_2$ = $\bar{\varphi}_3$ = 0 °.**

The selection of the initial dihedral angle for different creases greatly influences the stable state position of the origami element, which is the main factor that causes the structure to exhibit different deformation modes. Under certain initial dihedral angles of creases 1, 2, 3 and 4, the potential energy of the origami element first decreases and then increases as the height of the structure decreases during deformation as shown in Supplementary Fig. 14(a) and (b). Moreover, the structural height under the minimum potential energy varies with the initial dihedral angle. In these cases, the origami element reaches equilibrium at minimum potential energy, and the potential energy caused by creases' expansion

effect U_{exp} is equal to that caused by creases' contraction effect U_{con}. Under some initial dihedral angles, it is impossible to achieve equilibrium only through the forces of the creases. The geometry restriction at maximum structural height H_{max} results in the facets being in tension to counterweigh the extra expansion effect by the bending stress of creases as shown in Supplementary Fig. 14(c). The potential energy caused by creases' expansion effect U_{exp} is larger than that caused by creases' contraction U_{con}.

According to the relationship between the potential energy generated by the expansion and contraction effects, the response of origami element can be classified into two categories: complete-elastic and rigid-elastic responses. The complete-elastic response is mainly manifested as Mode I, Mode II and Mode III, and the rigid-elastic response is mainly manifested as the deformation of Mode IV. First, we investigate the classification of different responses under pairwise combinatorial examination of the initial dihedral angles, as shown in Supplementary Fig. 15, Supplementary Fig. 16, Supplementary Fig. 17, Supplementary Fig. 18, Supplementary Fig. 19 and Supplementary Fig. 20. It is found that (1) the

Supplementary Table 3: **The proportion of rigid-elastic response to all responses**

Proportion (%) / $\bar{\varphi}_2$ (°) / $\bar{\varphi}_4$ (°)	0	180	360
0	87.44	90.43	93.06
180	82.76	86.19	89.41
360	77.21	81.30	85.03

larger $\bar{\varphi}_2$ and $\bar{\varphi}_3$ and the smaller $\bar{\varphi}_1$ and $\bar{\varphi}_4$ make the expansion tendency of origami element stronger so that the structure is more likely to exhibit rigid-elastic response (pink region). Conversely, the structure is more likely to exhibit a complete-elastic response (light blue region and dark blue line). In addition, (2) the change in the initial dihedral angle of the creases with greater mechanical contribution (creases 1 and 3) has a greater impact on the structural response performance. (3) The boundary line distinguishing rigid-elastic and complete-elastic responses but classified as complete-elastic response always moves in the figure with the same slope for the same combination of two initial dihedral angle variables. When the difference of the mechanical contributions between two creases is greater, the boundary line tends to be perpendicular to the axis corresponding to the crease with a large mechanical contribution in the figures. Due to the major mechanical contribution of creases 1 and 3, the rigid-elastic response occupies 77.21% to 93.06% of the parameter space, as shown in Supplementary Table 3.

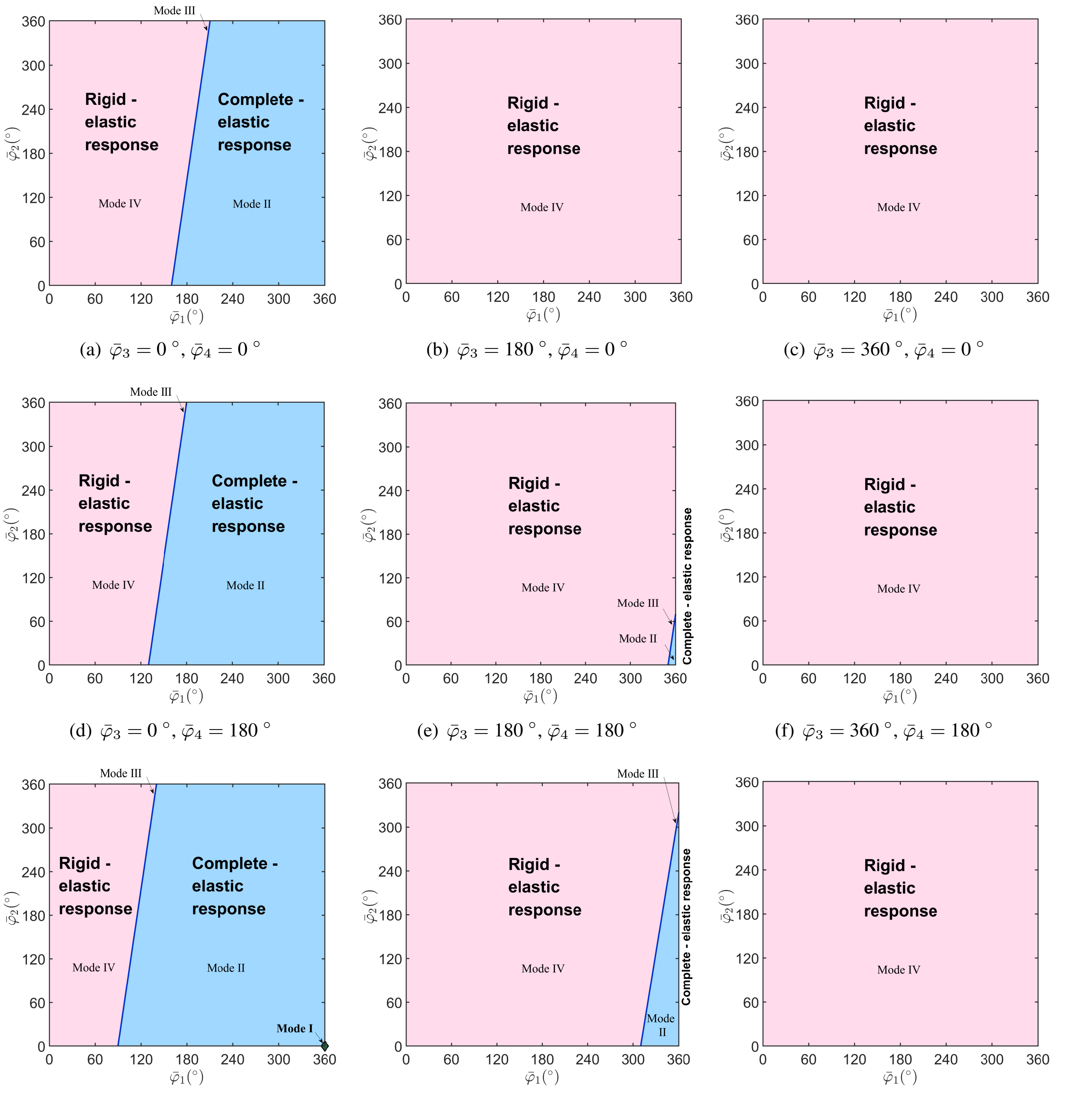

(a) $\bar{\varphi}_3 = 0\,^\circ$, $\bar{\varphi}_4 = 0\,^\circ$ (b) $\bar{\varphi}_3 = 180\,^\circ$, $\bar{\varphi}_4 = 0\,^\circ$ (c) $\bar{\varphi}_3 = 360\,^\circ$, $\bar{\varphi}_4 = 0\,^\circ$

(d) $\bar{\varphi}_3 = 0\,^\circ$, $\bar{\varphi}_4 = 180\,^\circ$ (e) $\bar{\varphi}_3 = 180\,^\circ$, $\bar{\varphi}_4 = 180\,^\circ$ (f) $\bar{\varphi}_3 = 360\,^\circ$, $\bar{\varphi}_4 = 180\,^\circ$

(g) $\bar{\varphi}_3 = 0\,^\circ$, $\bar{\varphi}_4 = 360\,^\circ$ (h) $\bar{\varphi}_3 = 180\,^\circ$, $\bar{\varphi}_4 = 360\,^\circ$ (i) $\bar{\varphi}_3 = 360\,^\circ$, $\bar{\varphi}_4 = 360\,^\circ$

Supplementary Fig. 15: **The classification of rigid-elastic and complete-elastic responses varied by the continuously changing initial dihedral angle $\bar{\varphi}_1$ and $\bar{\varphi}_2$ under fixed $\bar{\varphi}_3$ and $\bar{\varphi}_4$.** $\bar{\varphi}_3$ and $\bar{\varphi}_4$ are, respectively, fixed at (a) $0\,^\circ$ and $0\,^\circ$, (b) $180\,^\circ$ and $0\,^\circ$, (c) $360\,^\circ$ and $0\,^\circ$, (d) $0\,^\circ$ and $180\,^\circ$, (e) $180\,^\circ$ and $180\,^\circ$, (f) $360\,^\circ$ and $180\,^\circ$, (g) $0\,^\circ$ and $360\,^\circ$, (h) $180\,^\circ$ and $360\,^\circ$, (i) $360\,^\circ$ and $360\,^\circ$.

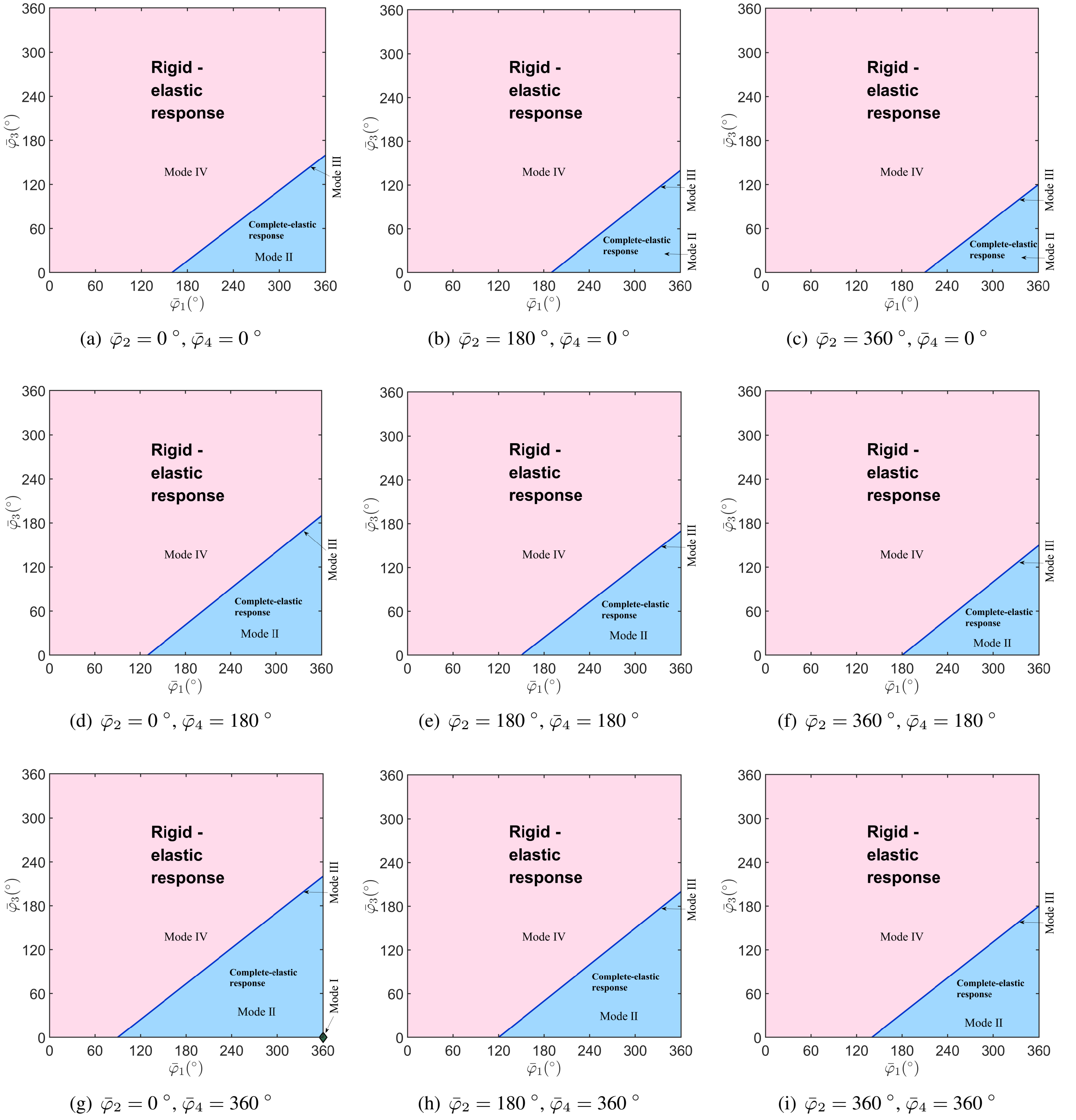

(a) $\bar{\varphi}_2 = 0\,^\circ$, $\bar{\varphi}_4 = 0\,^\circ$ (b) $\bar{\varphi}_2 = 180\,^\circ$, $\bar{\varphi}_4 = 0\,^\circ$ (c) $\bar{\varphi}_2 = 360\,^\circ$, $\bar{\varphi}_4 = 0\,^\circ$

(d) $\bar{\varphi}_2 = 0\,^\circ$, $\bar{\varphi}_4 = 180\,^\circ$ (e) $\bar{\varphi}_2 = 180\,^\circ$, $\bar{\varphi}_4 = 180\,^\circ$ (f) $\bar{\varphi}_2 = 360\,^\circ$, $\bar{\varphi}_4 = 180\,^\circ$

(g) $\bar{\varphi}_2 = 0\,^\circ$, $\bar{\varphi}_4 = 360\,^\circ$ (h) $\bar{\varphi}_2 = 180\,^\circ$, $\bar{\varphi}_4 = 360\,^\circ$ (i) $\bar{\varphi}_2 = 360\,^\circ$, $\bar{\varphi}_4 = 360\,^\circ$

Supplementary Fig. 16: **The classification of rigid-elastic and complete-elastic responses varied by the continuously changing initial dihedral angle $\bar{\varphi}_1$ and $\bar{\varphi}_3$ under fixed $\bar{\varphi}_2$ and $\bar{\varphi}_4$.** $\bar{\varphi}_2$ and $\bar{\varphi}_4$ are, respectively, fixed at (a) 0 ° and 0 °, (b) 180 ° and 0 °, (c) 360 ° and 0 °, (d) 0 ° and 180 °, (e) 180 ° and 180 °, (f) 360 ° and 180 °, (g) 0 ° and 360 °, (h) 180 ° and 360 °, (i) 360 ° and 360 °.

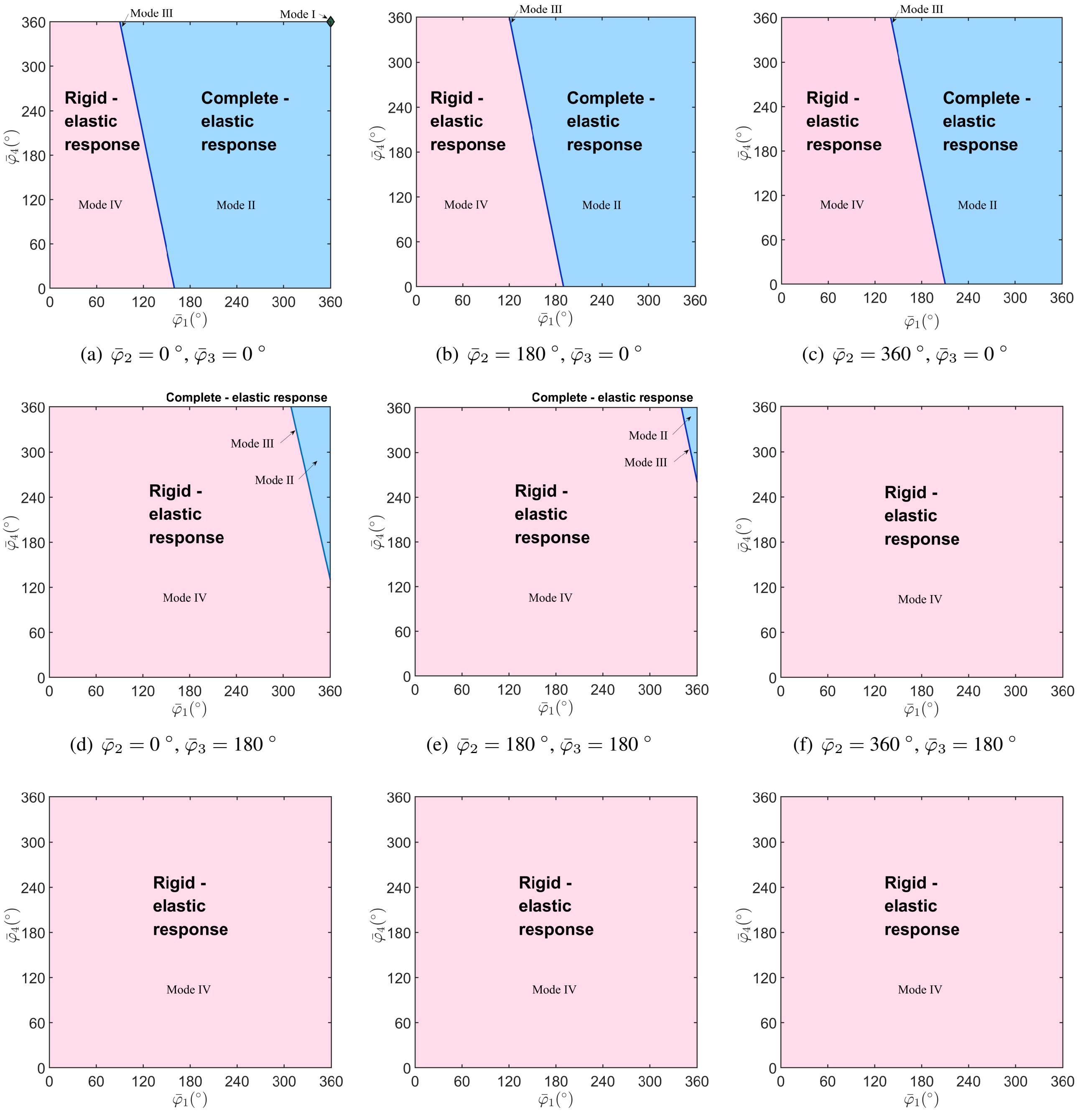

(a) $\bar{\varphi}_2 = 0\,^\circ, \bar{\varphi}_3 = 0\,^\circ$ (b) $\bar{\varphi}_2 = 180\,^\circ, \bar{\varphi}_3 = 0\,^\circ$ (c) $\bar{\varphi}_2 = 360\,^\circ, \bar{\varphi}_3 = 0\,^\circ$

(d) $\bar{\varphi}_2 = 0\,^\circ, \bar{\varphi}_3 = 180\,^\circ$ (e) $\bar{\varphi}_2 = 180\,^\circ, \bar{\varphi}_3 = 180\,^\circ$ (f) $\bar{\varphi}_2 = 360\,^\circ, \bar{\varphi}_3 = 180\,^\circ$

(g) $\bar{\varphi}_2 = 0\,^\circ, \bar{\varphi}_3 = 360\,^\circ$ (h) $\bar{\varphi}_2 = 180\,^\circ, \bar{\varphi}_3 = 360\,^\circ$ (i) $\bar{\varphi}_2 = 360\,^\circ, \bar{\varphi}_3 = 360\,^\circ$

Supplementary Fig. 17: **The classification of rigid-elastic and complete-elastic responses varied by the continuously changing initial dihedral angle $\bar{\varphi}_1$ and $\bar{\varphi}_4$ under fixed $\bar{\varphi}_2$ and $\bar{\varphi}_3$.** $\bar{\varphi}_2$ and $\bar{\varphi}_3$ are, respectively, fixed at (a) 0 ° and 0 °, (b) 180 ° and 0 °, (c) 360 ° and 0 °, (d) 0 ° and 180 °, (e) 180 ° and 180 °, (f) 360 ° and 180 °, (g) 0 ° and 360 °, (h) 180 ° and 360 °, (i) 360 ° and 360 °.

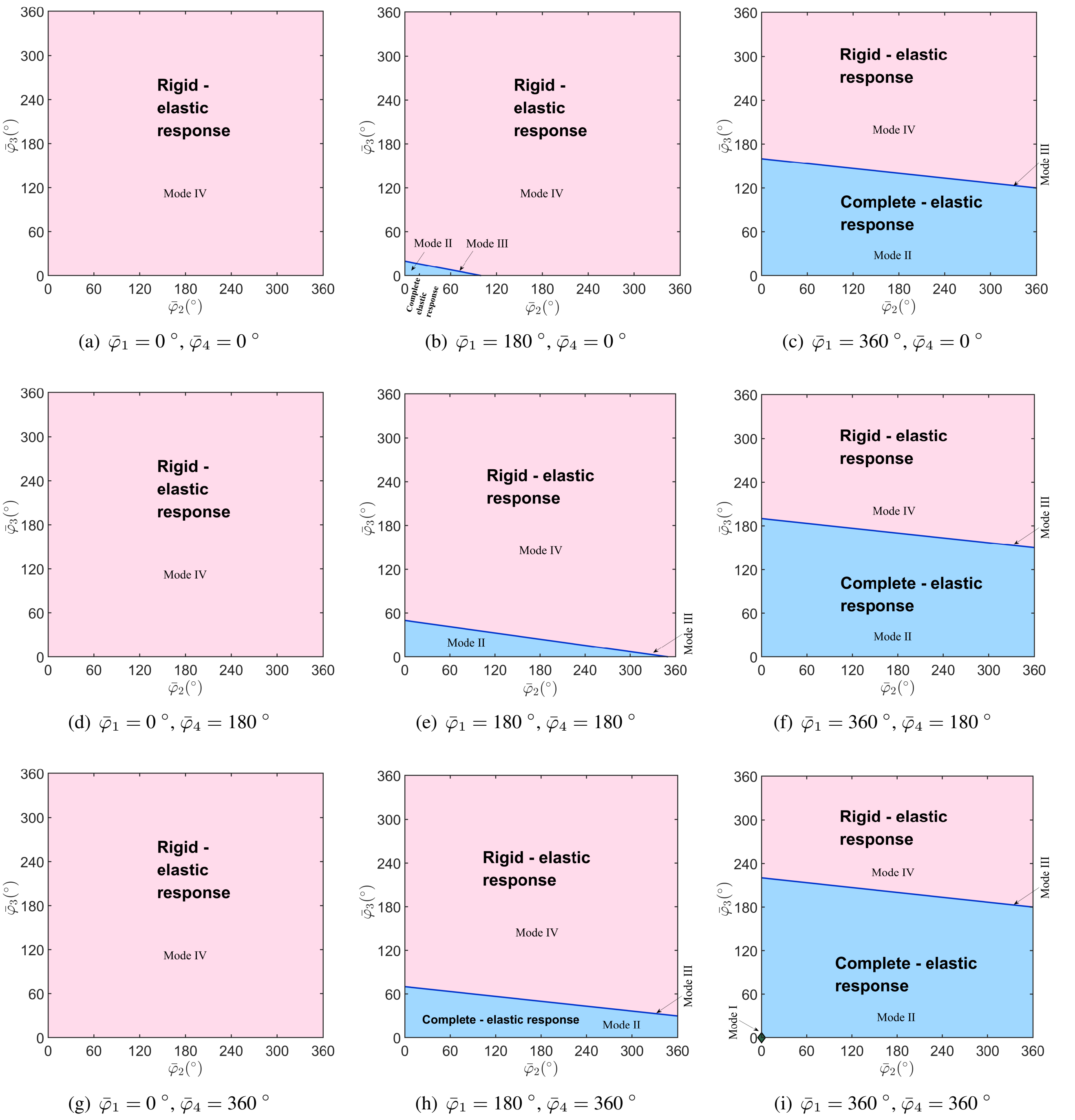

(a) $\bar{\varphi}_1 = 0\,^\circ$, $\bar{\varphi}_4 = 0\,^\circ$
(b) $\bar{\varphi}_1 = 180\,^\circ$, $\bar{\varphi}_4 = 0\,^\circ$
(c) $\bar{\varphi}_1 = 360\,^\circ$, $\bar{\varphi}_4 = 0\,^\circ$
(d) $\bar{\varphi}_1 = 0\,^\circ$, $\bar{\varphi}_4 = 180\,^\circ$
(e) $\bar{\varphi}_1 = 180\,^\circ$, $\bar{\varphi}_4 = 180\,^\circ$
(f) $\bar{\varphi}_1 = 360\,^\circ$, $\bar{\varphi}_4 = 180\,^\circ$
(g) $\bar{\varphi}_1 = 0\,^\circ$, $\bar{\varphi}_4 = 360\,^\circ$
(h) $\bar{\varphi}_1 = 180\,^\circ$, $\bar{\varphi}_4 = 360\,^\circ$
(i) $\bar{\varphi}_1 = 360\,^\circ$, $\bar{\varphi}_4 = 360\,^\circ$

Supplementary Fig. 18: **The classification of rigid-elastic and complete-elastic responses varied by the continuously changing initial dihedral angle $\bar{\varphi}_2$ and $\bar{\varphi}_3$ under fixed $\bar{\varphi}_1$ and $\bar{\varphi}_4$.** $\bar{\varphi}_1$ and $\bar{\varphi}_4$ are, respectively, fixed at (a) 0 ° and 0 °, (b) 180 ° and 0 °, (c) 360 ° and 0 °, (d) 0 ° and 180 °, (e) 180 ° and 180 °, (f) 360 ° and 180 °, (g) 0 ° and 360 °, (h) 180 ° and 360 °, (i) 360 ° and 360 °.

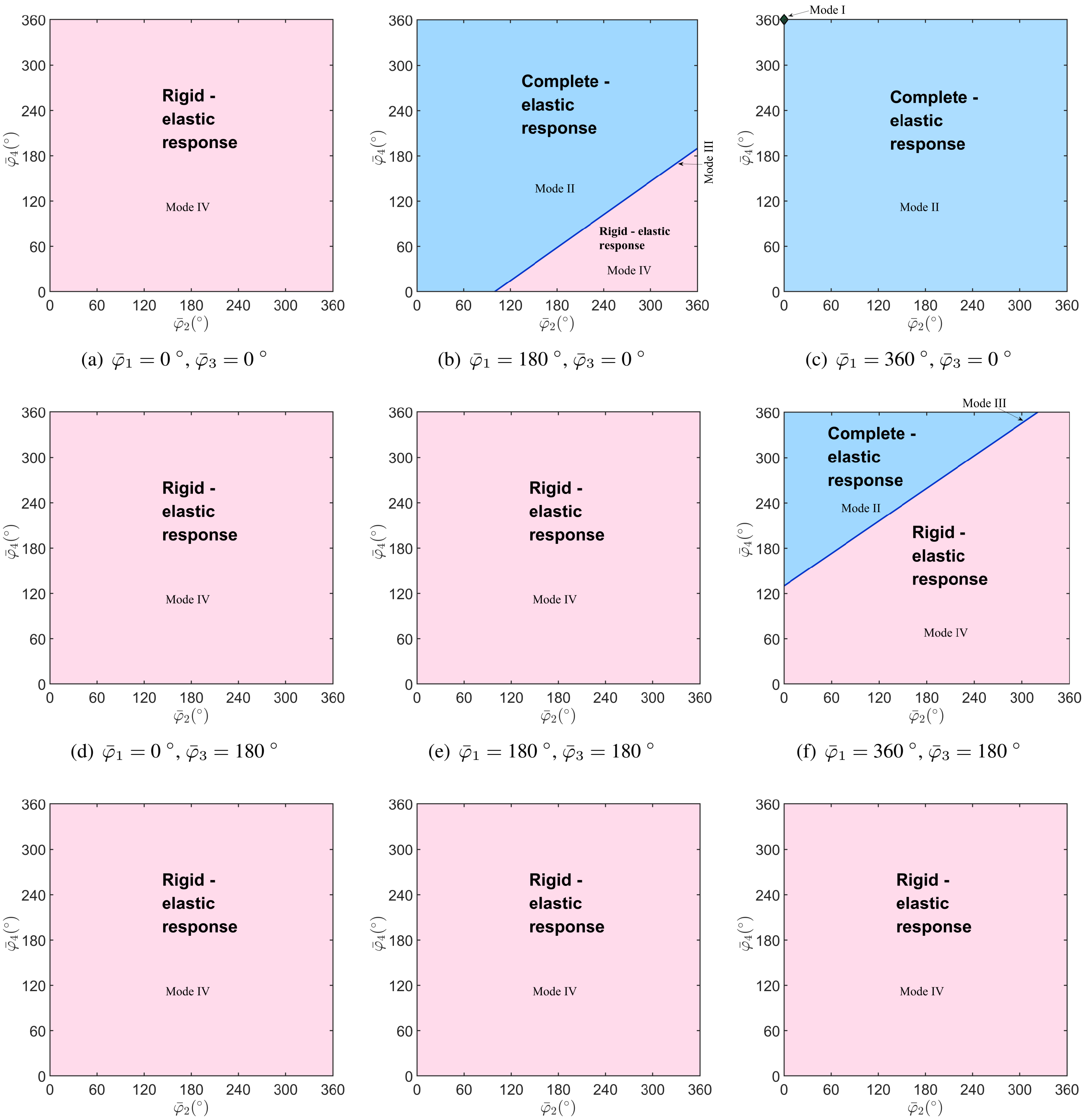

(a) $\bar{\varphi}_1 = 0\,^\circ$, $\bar{\varphi}_3 = 0\,^\circ$ (b) $\bar{\varphi}_1 = 180\,^\circ$, $\bar{\varphi}_3 = 0\,^\circ$ (c) $\bar{\varphi}_1 = 360\,^\circ$, $\bar{\varphi}_3 = 0\,^\circ$

(d) $\bar{\varphi}_1 = 0\,^\circ$, $\bar{\varphi}_3 = 180\,^\circ$ (e) $\bar{\varphi}_1 = 180\,^\circ$, $\bar{\varphi}_3 = 180\,^\circ$ (f) $\bar{\varphi}_1 = 360\,^\circ$, $\bar{\varphi}_3 = 180\,^\circ$

(g) $\bar{\varphi}_1 = 0\,^\circ$, $\bar{\varphi}_3 = 360\,^\circ$ (h) $\bar{\varphi}_1 = 180\,^\circ$, $\bar{\varphi}_3 = 360\,^\circ$ (i) $\bar{\varphi}_1 = 360\,^\circ$, $\bar{\varphi}_3 = 360\,^\circ$

Supplementary Fig. 19: **The classification of rigid-elastic and complete-elastic responses varied by the continuously changing initial dihedral angle $\bar{\varphi}_2$ and $\bar{\varphi}_4$ under fixed $\bar{\varphi}_1$ and $\bar{\varphi}_3$.** $\bar{\varphi}_1$ and $\bar{\varphi}_3$ are, respectively, fixed at (a) 0 ° and 0 °, (b) 180 ° and 0 °, (c) 360 ° and 0 °, (d) 0 ° and 180 °, (e) 180 ° and 180 °, (f) 360 ° and 180 °, (g) 0 ° and 360 °, (h) 180 ° and 360 °, (i) 360 ° and 360 °.

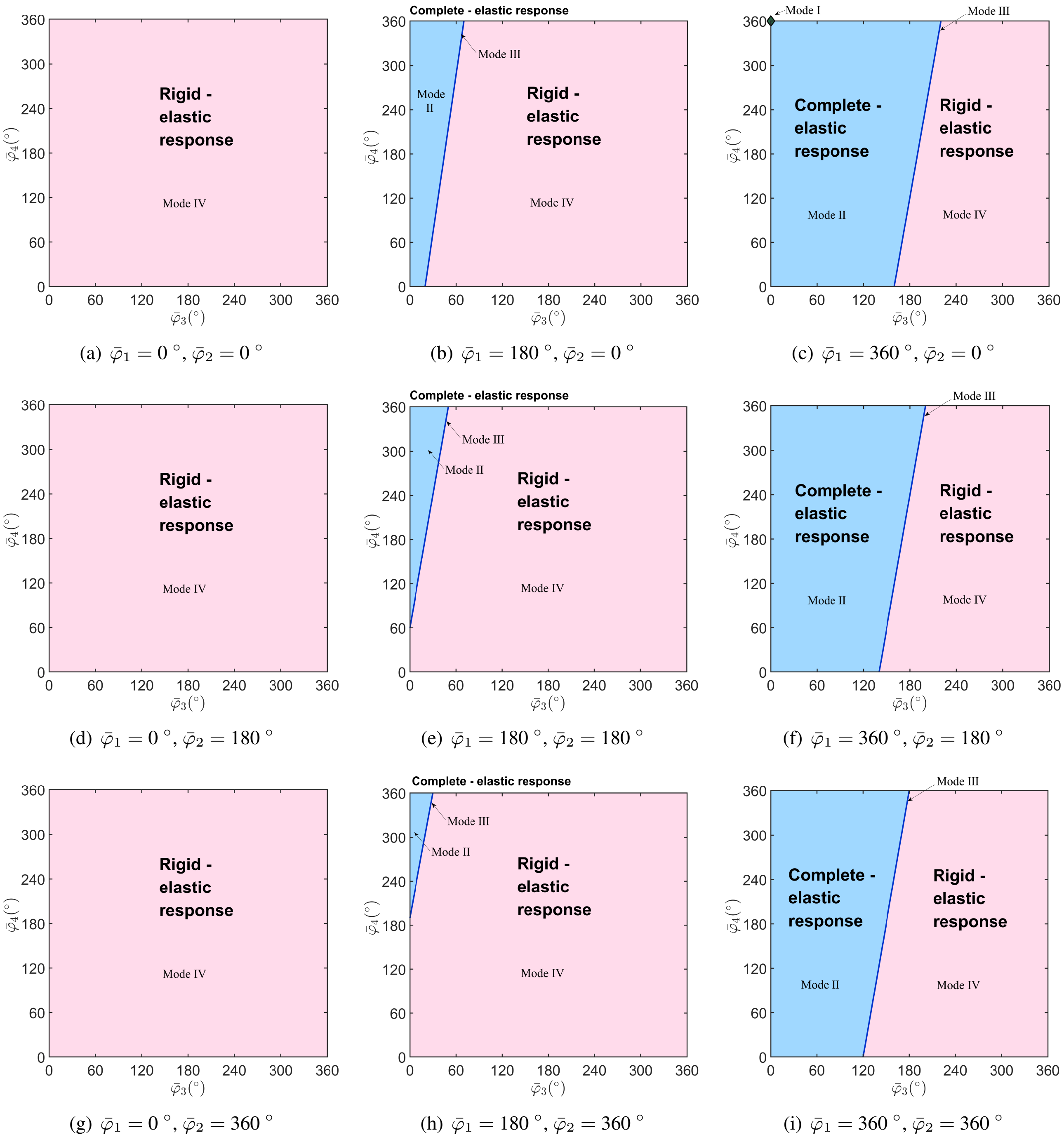

(a) $\bar{\varphi}_1 = 0\,^\circ$, $\bar{\varphi}_2 = 0\,^\circ$ (b) $\bar{\varphi}_1 = 180\,^\circ$, $\bar{\varphi}_2 = 0\,^\circ$ (c) $\bar{\varphi}_1 = 360\,^\circ$, $\bar{\varphi}_2 = 0\,^\circ$

(d) $\bar{\varphi}_1 = 0\,^\circ$, $\bar{\varphi}_2 = 180\,^\circ$ (e) $\bar{\varphi}_1 = 180\,^\circ$, $\bar{\varphi}_2 = 180\,^\circ$ (f) $\bar{\varphi}_1 = 360\,^\circ$, $\bar{\varphi}_2 = 180\,^\circ$

(g) $\bar{\varphi}_1 = 0\,^\circ$, $\bar{\varphi}_2 = 360\,^\circ$ (h) $\bar{\varphi}_1 = 180\,^\circ$, $\bar{\varphi}_2 = 360\,^\circ$ (i) $\bar{\varphi}_1 = 360\,^\circ$, $\bar{\varphi}_2 = 360\,^\circ$

Supplementary Fig. 20: **The classification of rigid-elastic and complete-elastic responses varied by the continuously changing initial dihedral angle $\bar{\varphi}_3$ and $\bar{\varphi}_4$ under fixed $\bar{\varphi}_1$ and $\bar{\varphi}_2$.** $\bar{\varphi}_1$ and $\bar{\varphi}_2$ are, respectively, fixed at (a) 0 ° and 0 °, (b) 180 ° and 0 °, (c) 360 ° and 0 °, (d) 0 ° and 180 °, (e) 180 ° and 180 °, (f) 360 ° and 180 °, (g) 0 ° and 360 °, (h) 180 ° and 360 °, (i) 360 ° and 360 °.

7. Force threshold of rigid-elastic response

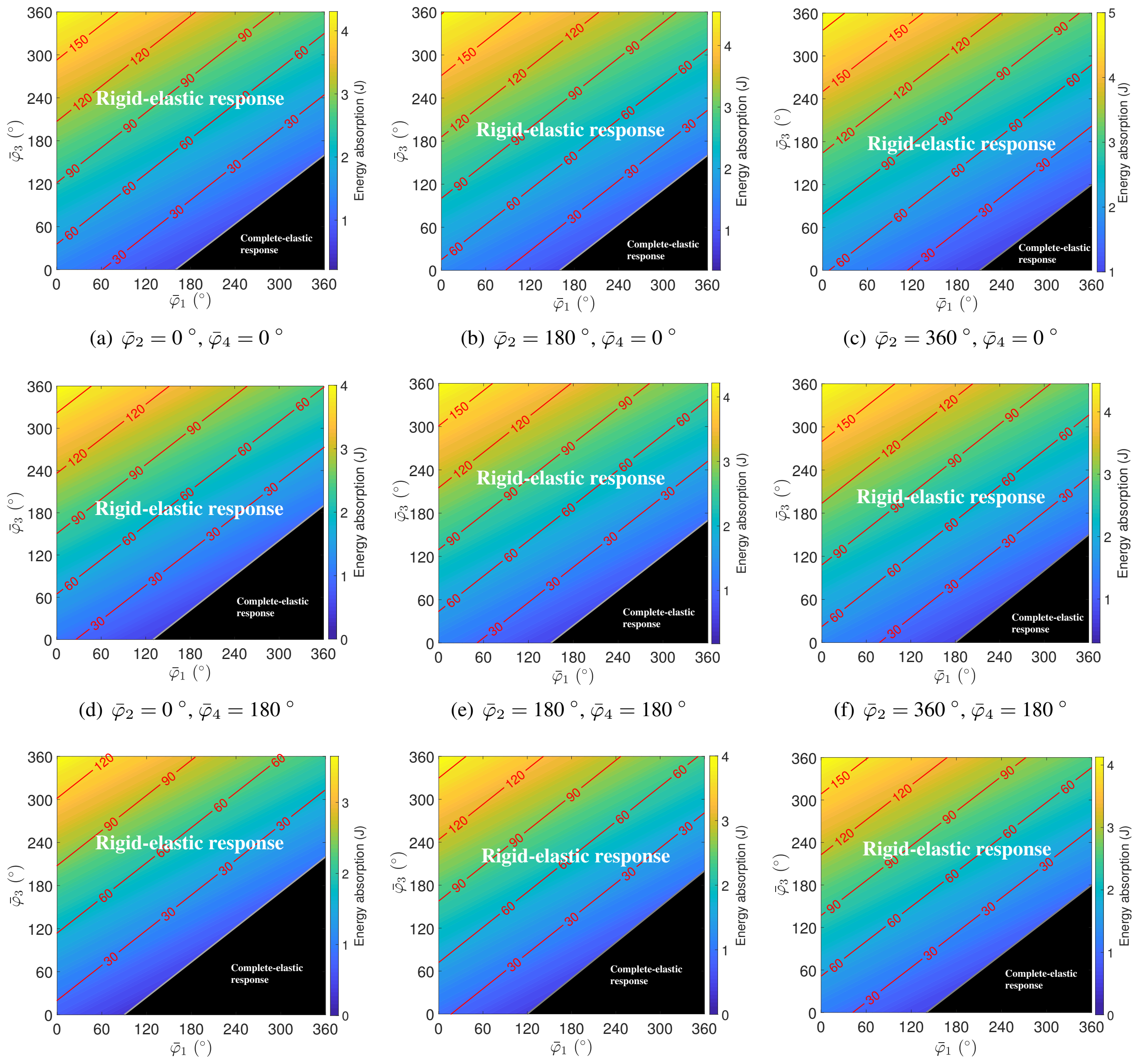

(a) $\bar{\varphi}_2 = 0\,°$, $\bar{\varphi}_4 = 0\,°$
(b) $\bar{\varphi}_2 = 180\,°$, $\bar{\varphi}_4 = 0\,°$
(c) $\bar{\varphi}_2 = 360\,°$, $\bar{\varphi}_4 = 0\,°$
(d) $\bar{\varphi}_2 = 0\,°$, $\bar{\varphi}_4 = 180\,°$
(e) $\bar{\varphi}_2 = 180\,°$, $\bar{\varphi}_4 = 180\,°$
(f) $\bar{\varphi}_2 = 360\,°$, $\bar{\varphi}_4 = 180\,°$
(g) $\bar{\varphi}_2 = 0\,°$, $\bar{\varphi}_4 = 360\,°$
(h) $\bar{\varphi}_2 = 180\,°$, $\bar{\varphi}_4 = 360\,°$
(i) $\bar{\varphi}_2 = 360\,°$, $\bar{\varphi}_4 = 360\,°$

Supplementary Fig. 21: **The potential energy change nephogram and force threshold contour (red lines) of origami element with rigid-elastic response under continuously changing initial dihedral angles $\bar{\varphi}_1$ and $\bar{\varphi}_3$ when $\bar{\varphi}_2$ and $\bar{\varphi}_4$ are fixed.** $\bar{\varphi}_2$ and $\bar{\varphi}_4$ are, respectively, fixed at (a) 0 ° and 0 °, (b) 180 ° and 0 °, (c) 360 ° and 0 °, (d) 0 ° and 180 °, (e) 180 ° and 180 °, (f) 360 ° and 180 °, (g) 0 ° and 360 °, (h) 180 ° and 360 °, (i) 360 ° and 360 °.

The initial dihedral angles of creases 1 and 3 with the maximum contribution factor among the mountain creases and valley creases, respectively, are key variables in the mechanical response programming of the origami. We program the initial dihedral angles $\bar{\varphi}_1$ and $\bar{\varphi}_3$ from 0° to 360° with $\bar{\varphi}_2$ of 360° and $\bar{\varphi}_4$ of 0° and observe that the rigid-elastic response dominates the parameter space (Supplementary Fig.21). The expansion effect is maximized by the mountain crease $\bar{\varphi}_1$ of 0° and the valley crease $\bar{\varphi}_3$ of 360°. Consequently, the force threshold of the rigid-elastic response and the changes in potential energy dependent on the expansion effect both increase with the decreasing $\bar{\varphi}_1$ and increasing $\bar{\varphi}_3$. Next, we analyze the proportion of the responses with force threshold by systematic sampling with a uniform interval of 15° for each initial dihedral angle. Among 390625 samples, the rigid-elastic response with static bifurcated full-length compression, the complete-elastic response with partial-length compression or tension, the complete-elastic response with full-length compression and the complete-elastic response with full-length tension occupy 85.72 %, 12.69 %, 1.59 % and 2.56×10^{-6} % of the four dimensional parameter space, respectively.

8. Stress-strain relationship and Poisson's ratio of reprogrammable mechanical metamaterials

The structural deformation of reprogrammable mechanical metamaterial is completed by deformation of building origami element in each functional-group. The change for each characteristic demension of the reprogrammable mechanical metamaterial has a certain inherent relationship with the height change of the deformable origami element. The shear stress or normal stress applied on the reprogrammable mechanical metamaterial is transmitted to each other along the splicing direction of the functional-groups. The gridling origami element always maintains a folding peoperty of pseudo single degree-of-freedom perpendicular at axial direction. Thus, the deformation of the functional-group can be measured by that of the contained origami element. When the origami elements in the reprogrammable mechanical metamaterial always deform cooperatively, the height variations for all origami elements are the same at any time. Therefore, the analytical solution for deformation characteristic dimension of the reprogrammable mechanical metamaterial and the reaction force can be calculated based on the geometric relationship.

A. Torsional deformation

If a point (o) on the functional-group, except for that at the hinge for connecting functional-groups, is taken as a fixed point, other points (a, b, c, a', b' and c') on the edge of the functional-group sweep a

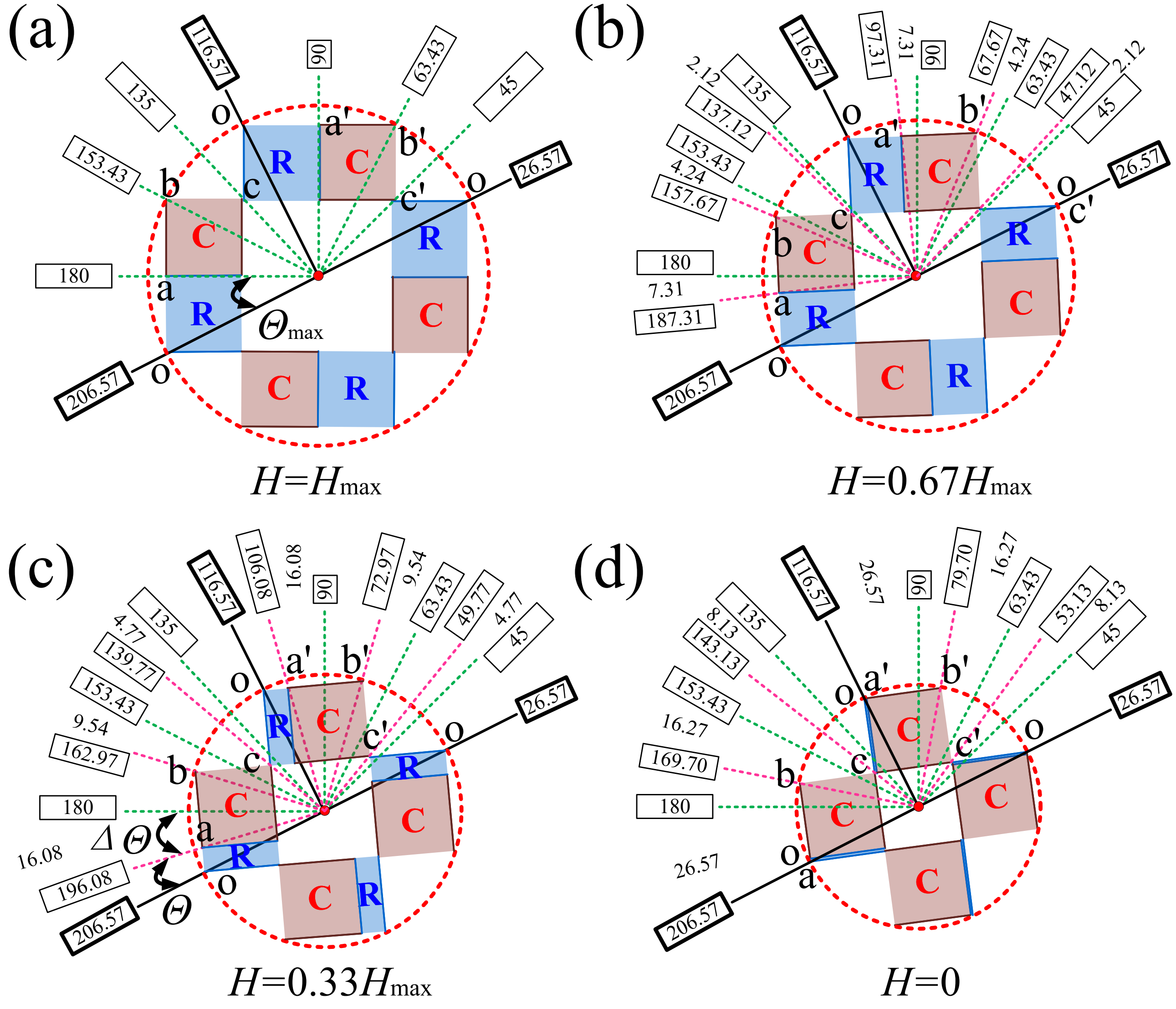

Supplementary Fig. 22: **Schematic diagram of torsional deformation of reprogrammable quadrilateral ring metamaterials and analysis of deformation angle processed by software Solidworks when the height of deformable origami element** H = **(a)** $H_{\max}$**, (b) 0.67**$H_{\max}$**, (c) 0.33**$H_{\max}$ **and (d) 0.** (Unit: °, the angle error is within 0.01 °)

certain angle around the central axis when all functional-groups in the reprogrammable mechanical metamaterial deform toward anticlockwise direction as Supplementary Fig. 22. Thus, the reprogrammable mechanical metamaterials exhibits torsional deformation. The torsional deformation is characterized by the different sweeping angles of different point in a single rotationally symmetric region. The sube-

qual ratio between the sweeping angles of different points in the deformation process through the angle analysis of software Solidworks.

For torsional deformation, the length of each side of the cavity triangle is always $H + H_{\max}$, such as side 1 in Supplementary Fig. 23(a), so the characteristic dimension $R_{\rm i}$ of the metamaterial can be expressed as

$$R_{\rm i} = \frac{H + H_{\max}}{2} \tan \frac{\pi(n_{\rm m} - 2)}{2n_{\rm m}} \tag{38}$$

Where $n_{\rm m}$ is the number of building functional-groups. The side length of the inner cavity triangle is the same as the length of parallel outer sides, such as side 1 and side 2. The distance between these two sides is always equal to $H_{\max}$. The characteristic dimension $R_{\rm o}$ of the reprogrammable mechanical metamaterial can be obtained by the sine theorem, which can be expressed for

$$R_{\rm o} = \sqrt{(R_{\rm i} + H_{\max})^2 + (H_{\max} + H)^2/4} \tag{39}$$

To analyse the change of structural feature $R_{\rm o}$ and $R_{\rm i}$ due to torsional deformation, we studied the

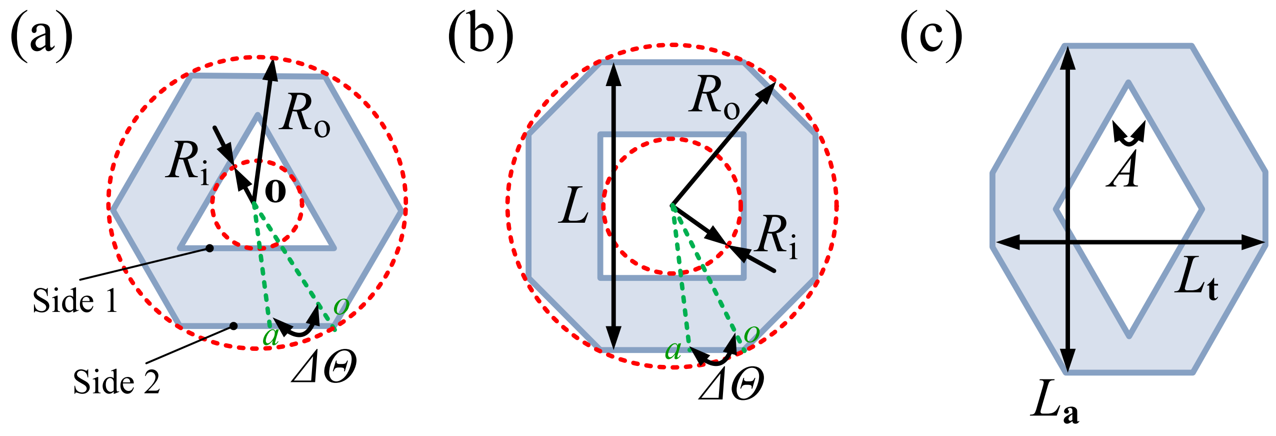

Supplementary Fig. 23: **Topological feature of reprogrammable mechanical metamaterial.** (a) triangular reprogrammable mechanical metamaterial. The (b) reprogrammable mechanical metamaterial and (c) its first morphology.

relationship between the sweeping angle of point a and $R_{\rm i}$. For determining the sweeping angle $\Delta\Theta$ at the point a of the reprogrammable mechanical metamaterial during deformation, we first calculate the maximum angle $\Theta_{\max}$ between the radius passing through point a (perpendicular to side 2) and the radius

passing through point o using the inverse triangle function

$$\Theta_{\max} = \tan^{-1}\frac{H_{\max}}{(R_{\mathrm{i}\max} + H_{\max})} \tag{40}$$

The current angle $\varTheta$ can be expressed as

$$\varTheta = \tan^{-1}\frac{H_{\max} + H}{2\,(R_{\mathrm{i}} + H_{\max})} - \tan^{-1}\frac{H_{\max} - H}{2\,(R_{\mathrm{i}} + H_{\max})} \tag{41}$$

So, the sweeping angle $\varDelta\varTheta$ is calculated as

$$\varDelta\varTheta = \varTheta_{\max} - \varTheta \tag{42}$$

The sweep angle of other points can be approximately obtained by multiplying the subequal ratio. In other words, $\varDelta\varTheta/\varDelta\varTheta_{\max}$ for any points is basically equal under any torsional deformation state.

The reaction force applied on the origami elements is assumed as F during folding or unfolding. Through the structural characteristics, we can determine the torque of the reprogrammable mechanical metamaterials during torsional deformation as

$$T = F\left(R_{\mathrm{i}} + \frac{H_{\max}}{2}\right) \tag{43}$$

The equivalent modulus to measure the difference of torsional deformation of reprogrammable mechanical metamaterials is obtained as

$$E_{\mathrm{e}} = \frac{dF}{(H_{\max})^2} \cdot \frac{R_{\mathrm{i}0}}{dR_{\mathrm{i}}} \tag{44}$$

B. Axial deformation with Poisson's ratio of 0

For axial deformation of reprogrammable quadrilateral ring metamaterials as shown in Fig.4, its deformation is determined by co-deformation of origami elements in non-adjacent building functional-groups. Therefore, the relationship between the characteristic dimension L for the reprogrammable quadrilateral ring metamaterials and the height of the deformable origami element in this case is

$$L = 3H_{\max} + H \tag{45}$$

Through the structural characteristics of metamaterials, we can calculate the concentrated force as following

$$F_1 = 2F \tag{46}$$

The reprogrammable mechanical metamaterial is considered as a block material with the occupied space of $L_{\text{stable}} \times L_{\text{stable}} \times H_{\max}$, and the initial cross section area of the specimen is considered as the maximum cross section area of the reprogrammable mechanical metamaterial [4]. The engineering compression stress is determined as

$$\sigma_1 = \frac{F_1}{(3H_{\max} + H_{\text{stable}})H_{\max}} \tag{47}$$

Since the reprogrammable mechanical metamaterial does not deform at one of the orthogonal directions, the Poisson's ratio in this case is 0.

C. Auxetic deformation

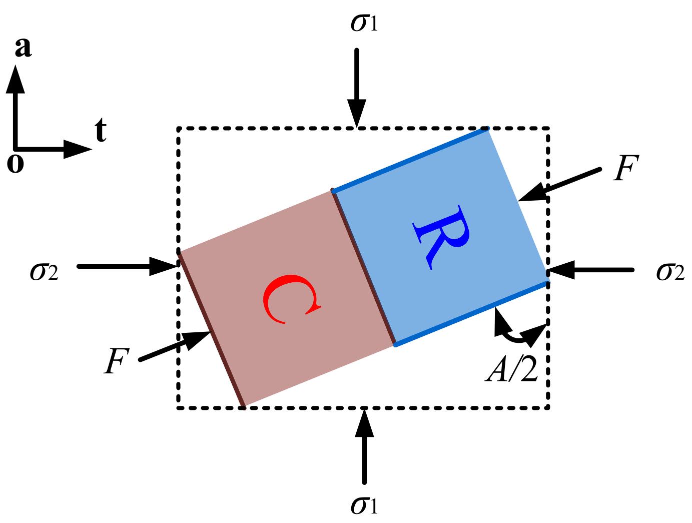

Supplementary Fig. 24: **representative volume element of periodic homogeneous quadrilateral ring metamaterials.**

For auxetic deformation for reprogrammable quadrilateral ring metamaterials as shown in Fig.4 and Fig.5, origami elements with the same mechanical response deform cooperatively. Although this Metamaterial has strong nonlinear mechanical properties, its mechanical response under small deformation can be considered as linear. In addition, judging from the structural deformation, the Poisson's ratio of

the Metamaterial is constant at a fixed adjustment angle, so we can calculate the material properties, such as Poisson's ratio, of the Metamaterial under small structural deformation. Therefore, we can use the constitutive relationship to reflect the inherent properties of metamaterials [5, 6]. For the metamaterial in this article, the constitutive relationship can be expressed as

$$\varepsilon_i = S_{ii}\sigma_i \Rightarrow \left\{ \begin{array}{c} \varepsilon_1 \\ \varepsilon_2 \end{array} \right\} = \left[\begin{array}{cc} S_{11} & S_{12} \\ sym & S_{22} \end{array} \right] \left\{ \begin{array}{c} \sigma_1 \\ \sigma_2 \end{array} \right\} \tag{48}$$

ε and σ are strain and stress of metamaterials, respectively. i=1,2, which represents the **a** and **t** directions, respectively, as shown in Supplementary Fig. 24. **S** is the compliance tensor. The functional-group is considered as representative volume element. The length of representative volume element at **a** direction is calculated by

$$L_{\mathbf{a}} = (H_{\max} + H)\cos\left(\frac{A}{2}\right) + H_{\max}\sin\frac{A}{2} \tag{49}$$

where A is the internal angle of reprogrammable mechanical metamaterials with quadrilateral ring. A = $\mathit{\Gamma}_i$ when reprogrammable mechanical metamaterial is transformed to C ring metamaterials, and A = $\mathit{\Omega}_i$ when reprogrammable mechanical metamaterial is transformed to R ring metamaterials. The length of representative volume element at **t** direction is calculated by

$$L_{\mathbf{t}} = (H_{\max} + H)\sin\left(\frac{A}{2}\right) + H_{\max}\cos\frac{A}{2} \tag{50}$$

When the metamaterial undergoes deformation, the boundary conditions of the representative volume element are

$$\begin{cases} \sigma_1 = F\cos(\dfrac{A}{2})\Big/(H_{\max}L_{\mathbf{t}}) \\ \sigma_2 = F\sin(\dfrac{A}{2})\Big/(H_{\max}L_{\mathbf{a}}) \end{cases} \tag{51}$$

The concentrated force of metamaterial is

$$\begin{cases} F_{\mathbf{a}} = mF\cos(\dfrac{A}{2}) \\ F_{\mathbf{t}} = nF\sin(\dfrac{A}{2}) \end{cases} \tag{52}$$

Where m and n are the number of representative volume element at **t** and **a** direction, respectively.

Similarly, the engineering stress is calculated as

$$\begin{cases} \sigma_{\mathbf{a}} = mF\cos(\dfrac{A}{2})\Big/(H_{\max}L_{\mathbf{t}}) \\ \sigma_{\mathbf{t}} = nF\sin(\dfrac{A}{2})\Big/(H_{\max}L_{\mathbf{a}}) \end{cases} \tag{53}$$

Assuming that the deformation of the representative volume element is δl, the strain energy density of representative volume element is determined as

$$U = k\delta l^2/2V \tag{54}$$

where k is the elements' stiffness under small deformation. V is the volume in which the potential energy U is considered. The components of the compliance tensor can be calculated [7] as

$$S_{11} = \frac{2U}{\sigma_1\sigma_1}, S_{12} = S_{21} = \frac{2U}{\sigma_1\sigma_2}, S_{22} = \frac{2U}{\sigma_2\sigma_2} \tag{55}$$

In addition, the components of compliance tensor can also be represented [8] as

$$S_{11} = \frac{1}{E_1}, S_{12} = S_{21} = -\frac{\nu_{12}}{E_1} = -\frac{\nu_{21}}{E_2}, S_{22} = \frac{1}{E_2} \tag{56}$$

Thus, the Poisson's ratios are defined as

$$\nu_{\mathbf{ta}} = -\frac{\sin(A/2)\left[H_{\max}\left(\cos(A/2)+\sin(A/2)\right)+H_{\text{stable}}\cos(A/2)\right]}{\cos(A/2)\left[H_{\max}\left(\cos(A/2)+\sin(A/2)\right)+H_{\text{stable}}\sin(A/2)\right]} \tag{57}$$

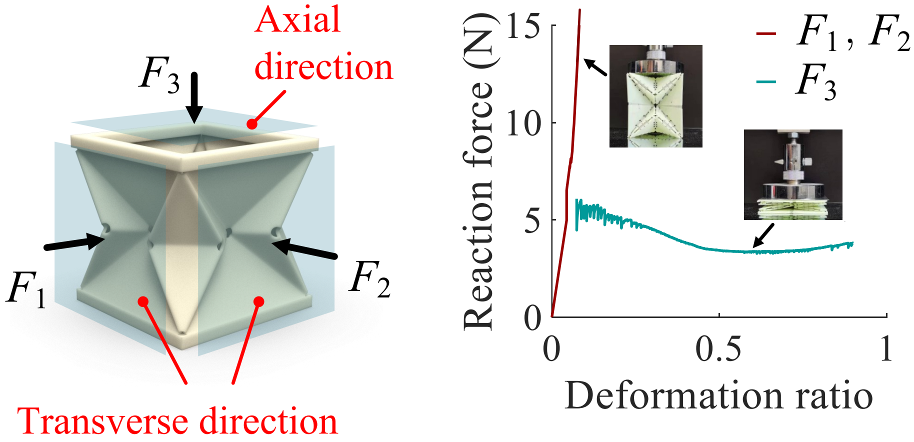

Supplementary Fig. 25: **Schematic diagram of forces acting on origami element in different directions and the experimental results.** Source data are provided as a Source Data file.

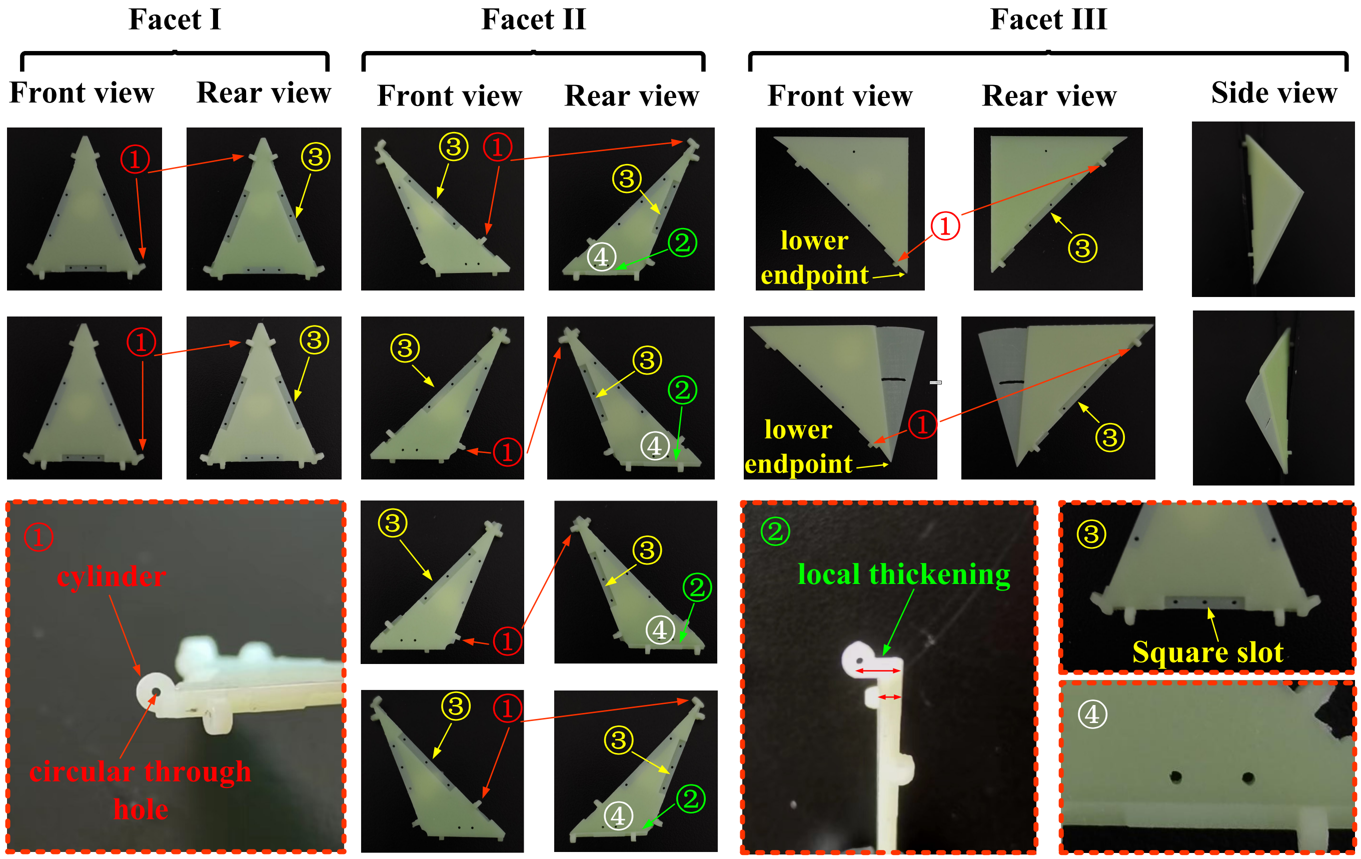

Supplementary Fig. 26: 3D printing products and the detailing display of facet I, facet II and facet III for origami element with high stiffness facets. ① The cylinder on the two connected facets can compose a rotating hinge, replacing crease bending in folding or unfolding, in pairs when two through holes remain concentric. ② locally thickeness of facet II. ③ Square slot is provided on each edge of each facet for mounting elastic creases 1, 2 and 3. ④ For crease 4 on facets II, screw holes are provided directly on rigid facet II to secure the crease material.

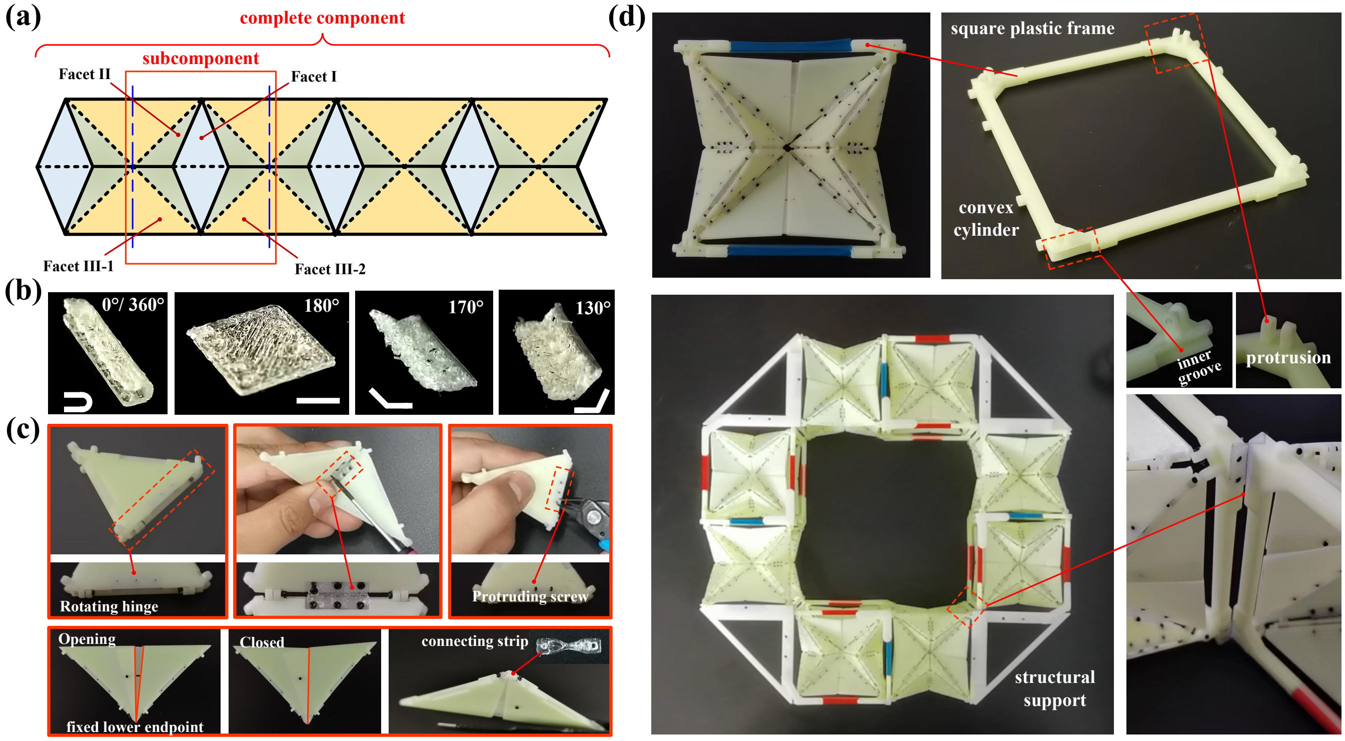

Supplementary Fig. 27: **Schematic Diagram of origmai element assembly and metamaterial assembly.** (a) Subcomponent and complete component of origami element. (b) 3D printed TPU creases. (c) Schematic diagram of hinge assembly, crease assembly, facet III assembly and surplus screw processing. (d) Schematic diagram of origami element connection for functional-groups construction and functional-group connection for metamaterials construction.

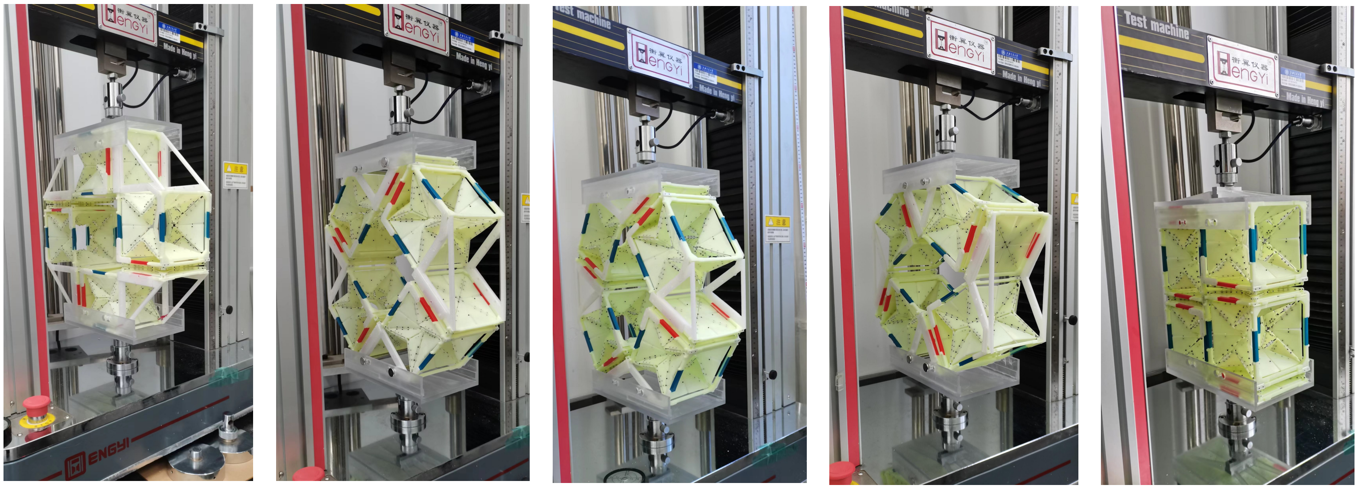

Supplementary Fig. 28: **Uniaxial mechanical tests of reprogrammable mechanical metamaterials.** The measured deformations from left to right are axial deformation with Poisson's ratio of 0, axial deformation with Poisson's ratio of - 1, deformation in the long axis direction under the first evolutionary morphology, deformation in the short axis direction under the first evolutionary morphology and deformation in the second evolutionary morphology.

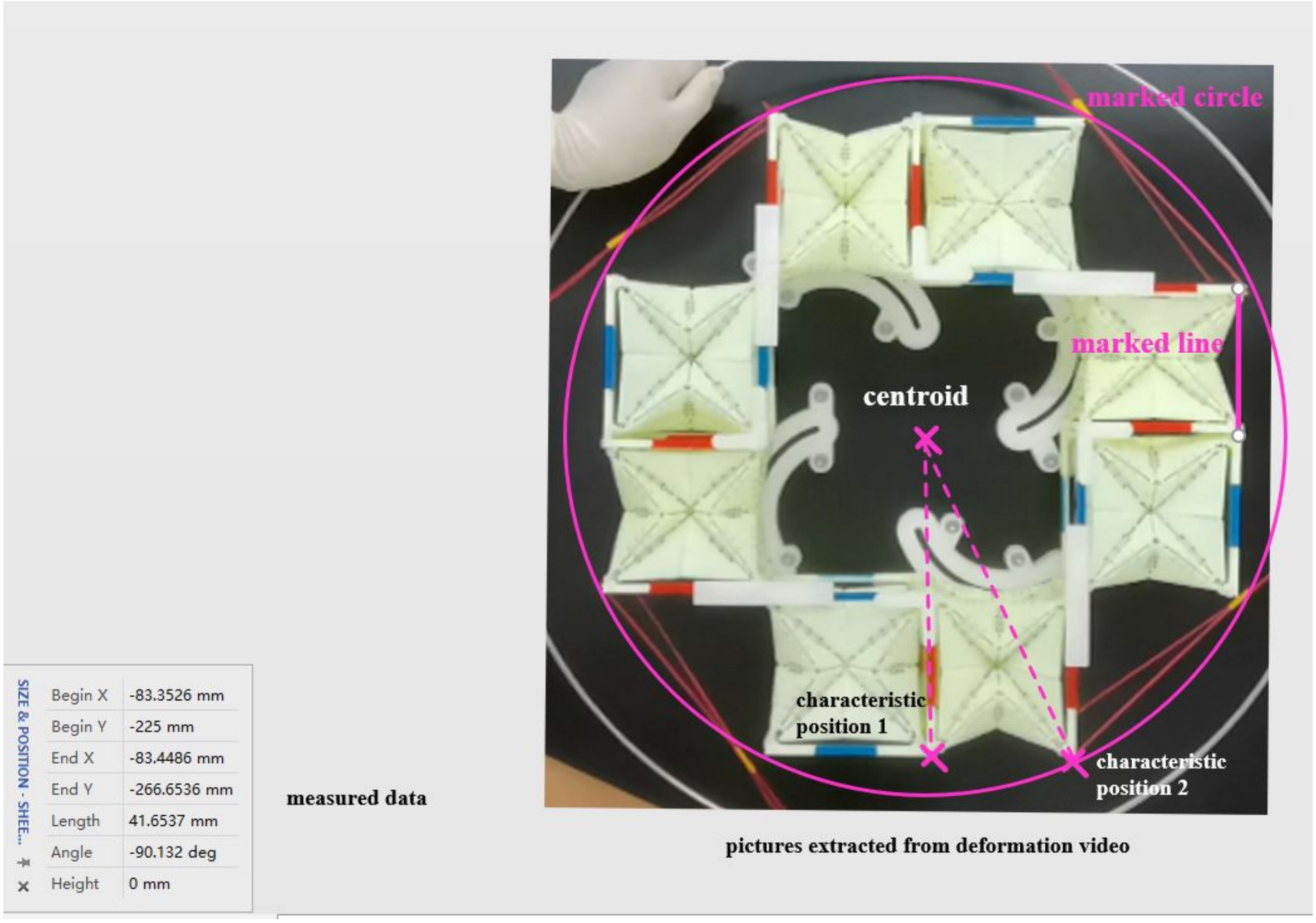

Supplementary Fig. 29: **Schematic diagram of digital image processing for torsion deformation.** The magenta lines in the figure are the auxiliary line for data extraction, and the lower left corner is the data extraction window.

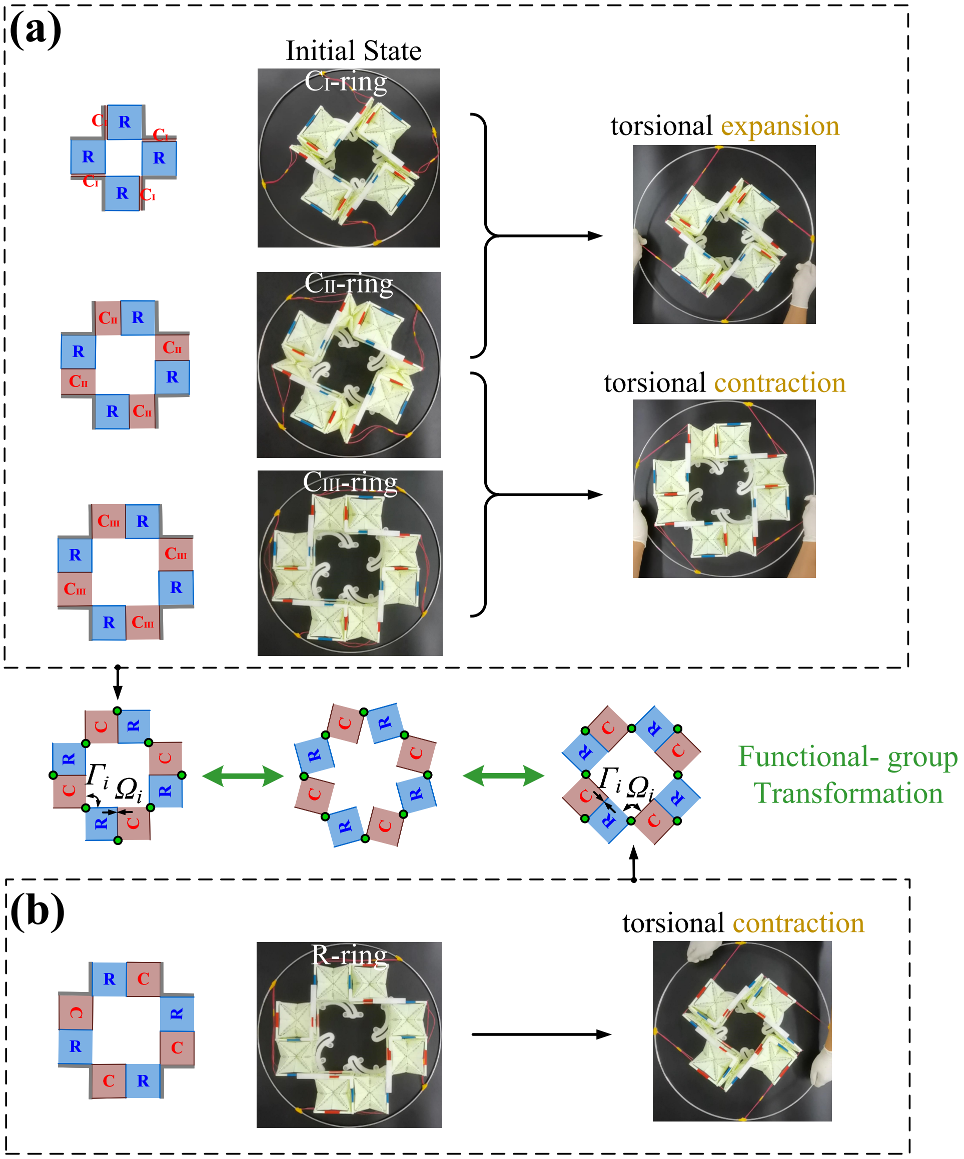

Supplementary Fig. 30: **Physical demonstration of torsion deformation of quadrilateral ring metamaterials with adjustable angle of π/2.** (a) The torsional deformation of quadrilateral ring metamaterials with C_I,C_{II} and C_{III} functional-groups. (b) The torsional deformation of quadrilateral ring metamaterials with R functional-groups.

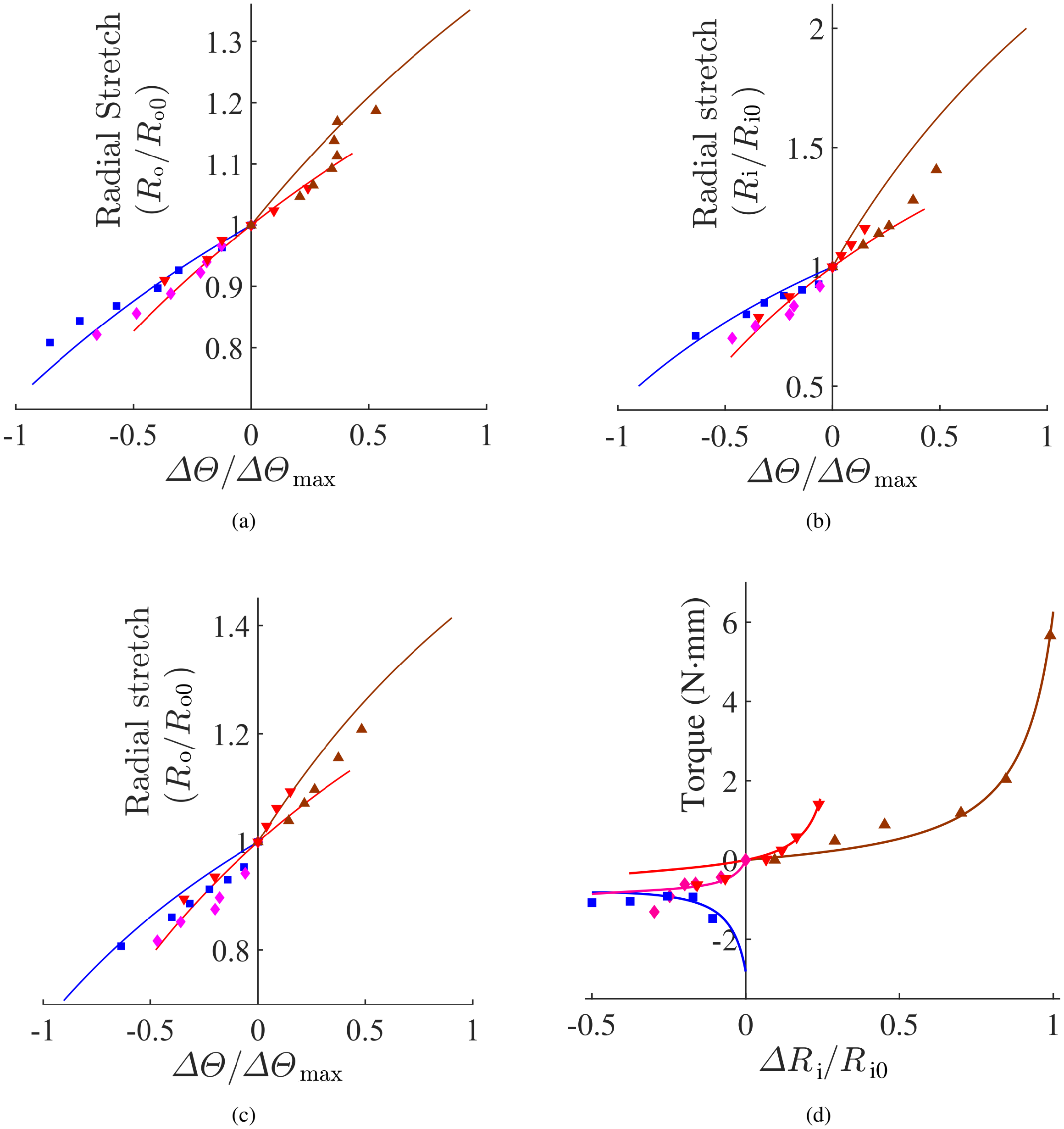

Supplementary Fig. 31: **The torsional deformation and mechanical characterization of reprogrammable mechanical metamaterials.** (a). Radial stretch R_o/R_{o0} varied as a function of $\Delta\Theta/\Delta\Theta_{max}$ of sweeping angle for reprogrammable triangular ring metamaterials. Source data are provided as a Source Data file. (b) Radial stretch R_i/R_{i0} and (c) Radial stretch R_o/R_{o0} varied as a function of $\Delta\Theta/\Delta\Theta_{max}$ of sweeping angle for reprogrammable quadrilateral ring metamaterials. The solid lines represent theoretical results, and the discrete points denote experimental results. Source data are provided as a Source Data file. (d) Theoretical and semi-experimental torque-radial strain results of reprogrammable quadrilateral ring metamaterials.

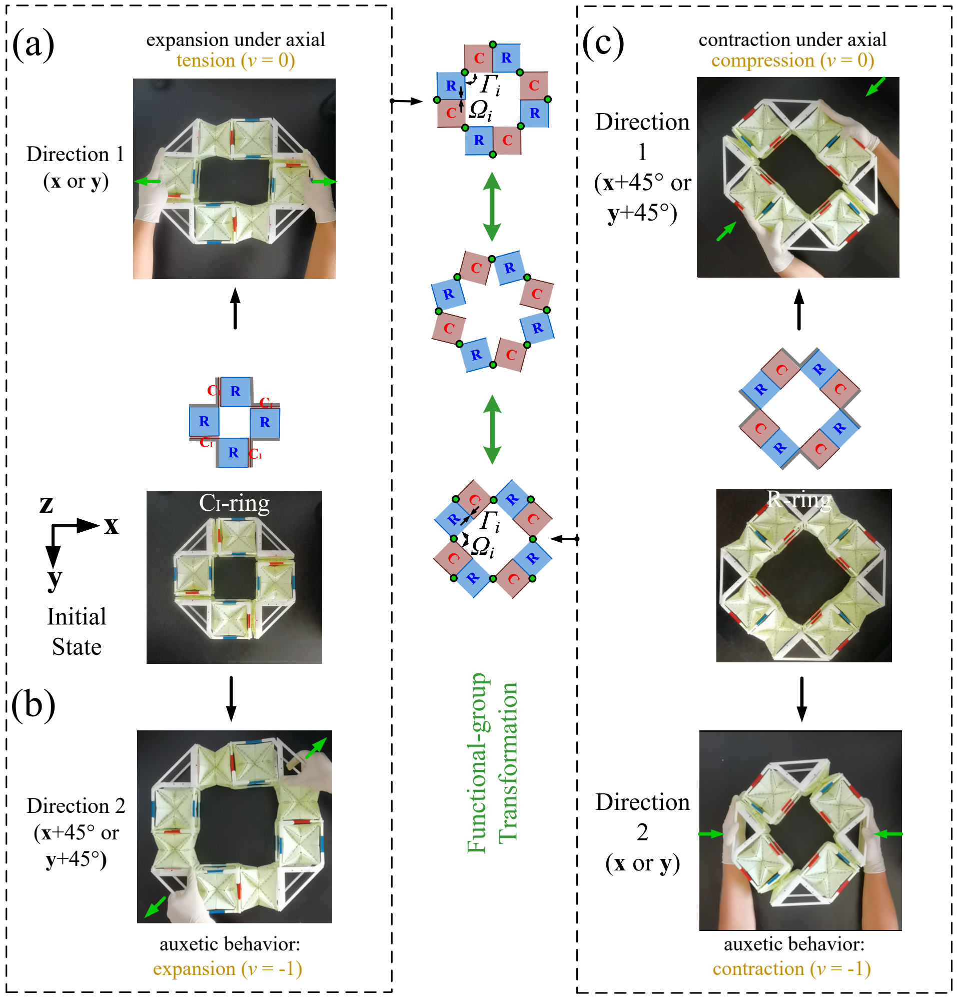

Supplementary Fig. 32: **Prototype snapshots of multidirectional deformation of quadrilateral ring metamaterials with** C_I **functional-groups.** (a) The expansion deformation of quadrilateral ring metamaterials with C_I functional-groups under axial tension and at direction 1. (b) The expansion deformation of quadrilateral ring metamaterials with C_I functional-groups under axial tension and at direction 2. (c) The contraction deformation of quadrilateral ring metamaterials with R functional-groups, which is transformed from quadrilateral ring metamaterials with C_I functional-groups, under axial tension and at direction 1 and 2.

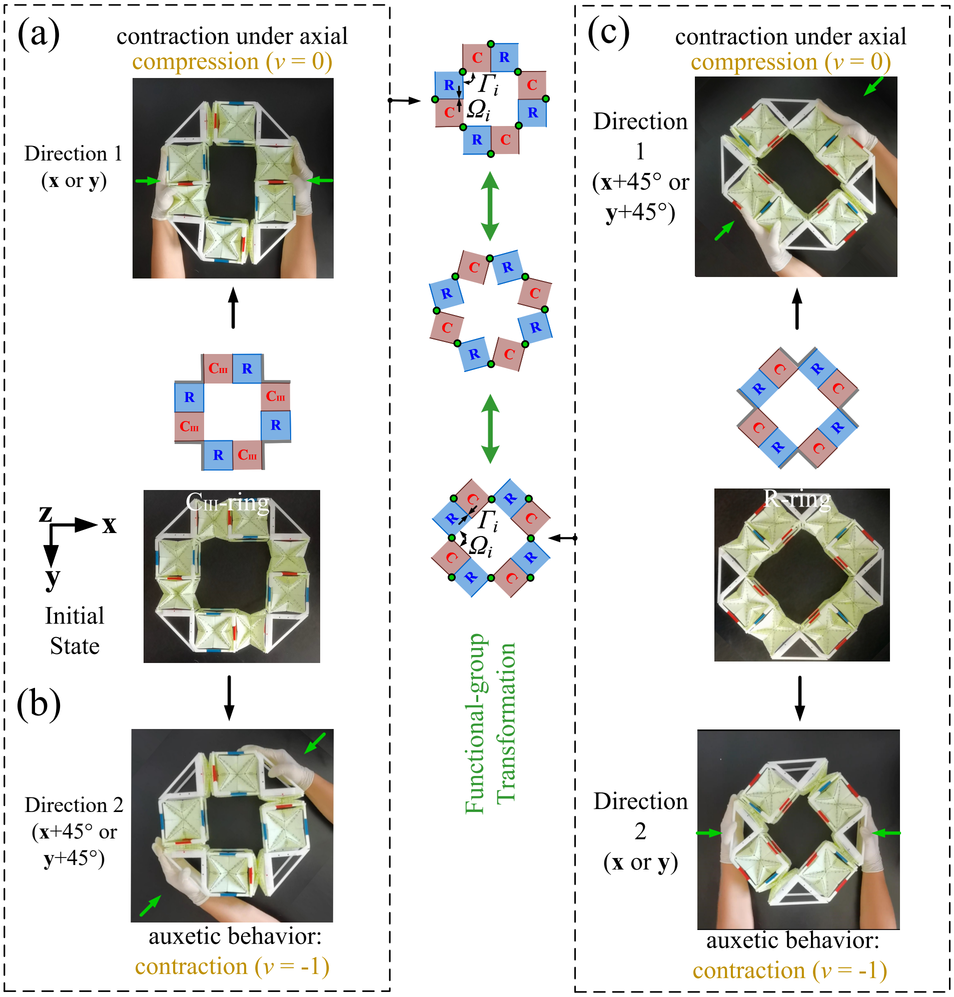

Supplementary Fig. 33: **Prototype snapshots of multidirectional deformation of quadrilateral ring metamaterials with** C_{III} **functional-groups.** (a) The contraction deformation of quadrilateral ring metamaterials with C_{III} functional-groups under axial tension and at direction 1. (b) The contraction deformation of quadrilateral ring metamaterials with C_{III} functional-groups under axial tension and at direction 2. (c) The contraction deformation of quadrilateral ring metamaterials with R functional-groups, which is transformed from quadrilateral ring metamaterials with C_{III} functional-groups, under axial tension and at direction 1 and 2.

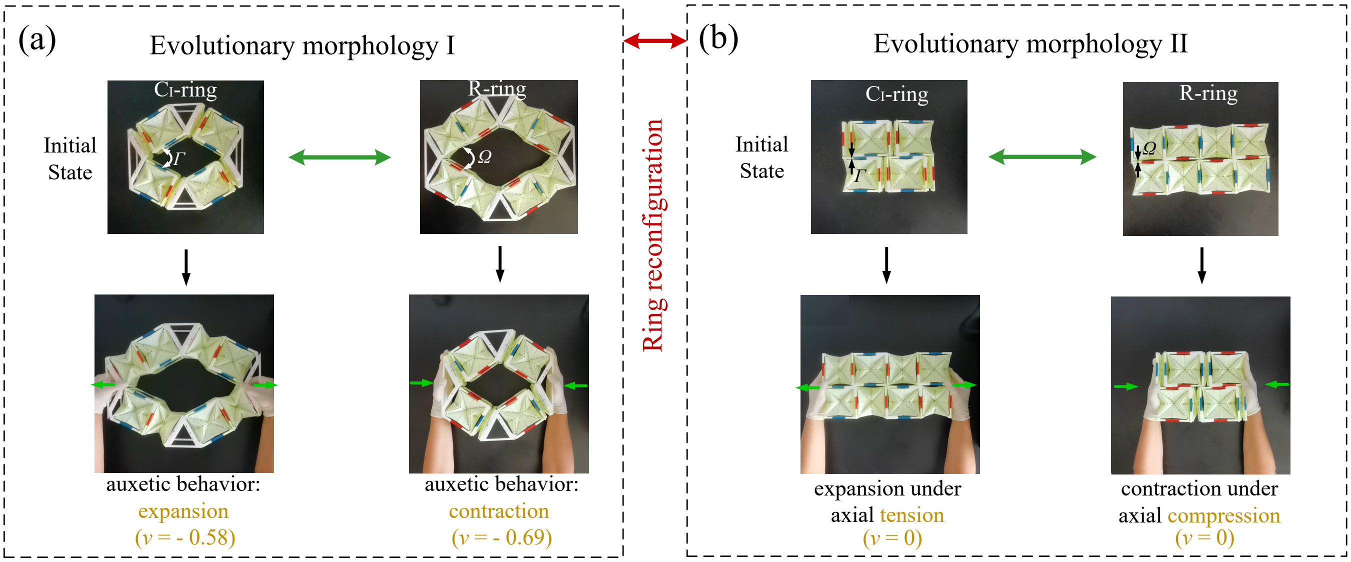

Supplementary Fig. 34: **Prototype snapshots of axial deformation of quadrilateral ring metamaterials under two evolutionary morphology.** (a) The expansion of auxetic behavior of quadrilateral ring metamaterial with C_I functional-groups, and the contraction of auxetic behavior of transformed quadrilateral ring metamaterial with R functional-groups under evolutionary morphology I. (b) The expansion deformation under axial tension of quadrilateral ring metamaterial with C_I functional-groups, and the contraction under axial compression of transformed quadrilateral ring metamaterial with R functional-groups under evolutionary morphology II.

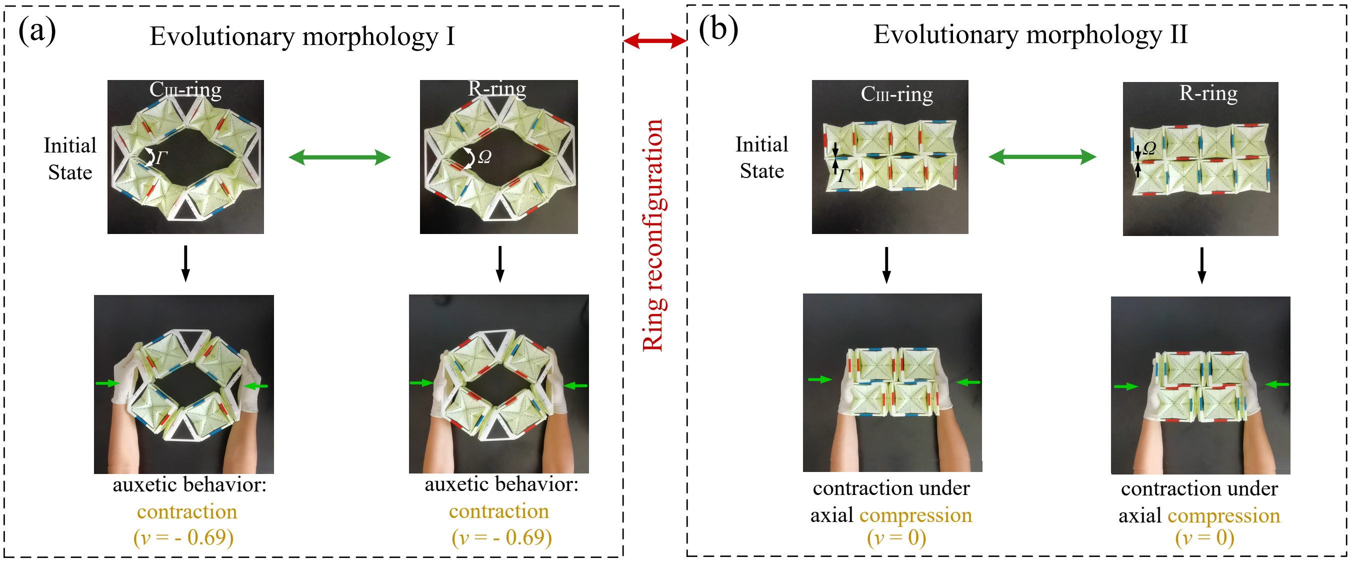

Supplementary Fig. 35: **Prototype snapshots of axial deformation of quadrilateral ring metamaterials under two evolutionary morphology.** (a) The contraction of auxetic behavior of quadrilateral ring metamaterial with C_{III} functional-groups, and the contraction of auxetic behavior of transformed quadrilateral ring metamaterial with R functional-groups under evolutionary morphology I. (b) The contraction deformation under axial tension of quadrilateral ring metamaterial with C_{III} functional-groups, and the contraction under axial compression of transformed quadrilateral ring metamaterial with R functional-groups under evolutionary morphology II.

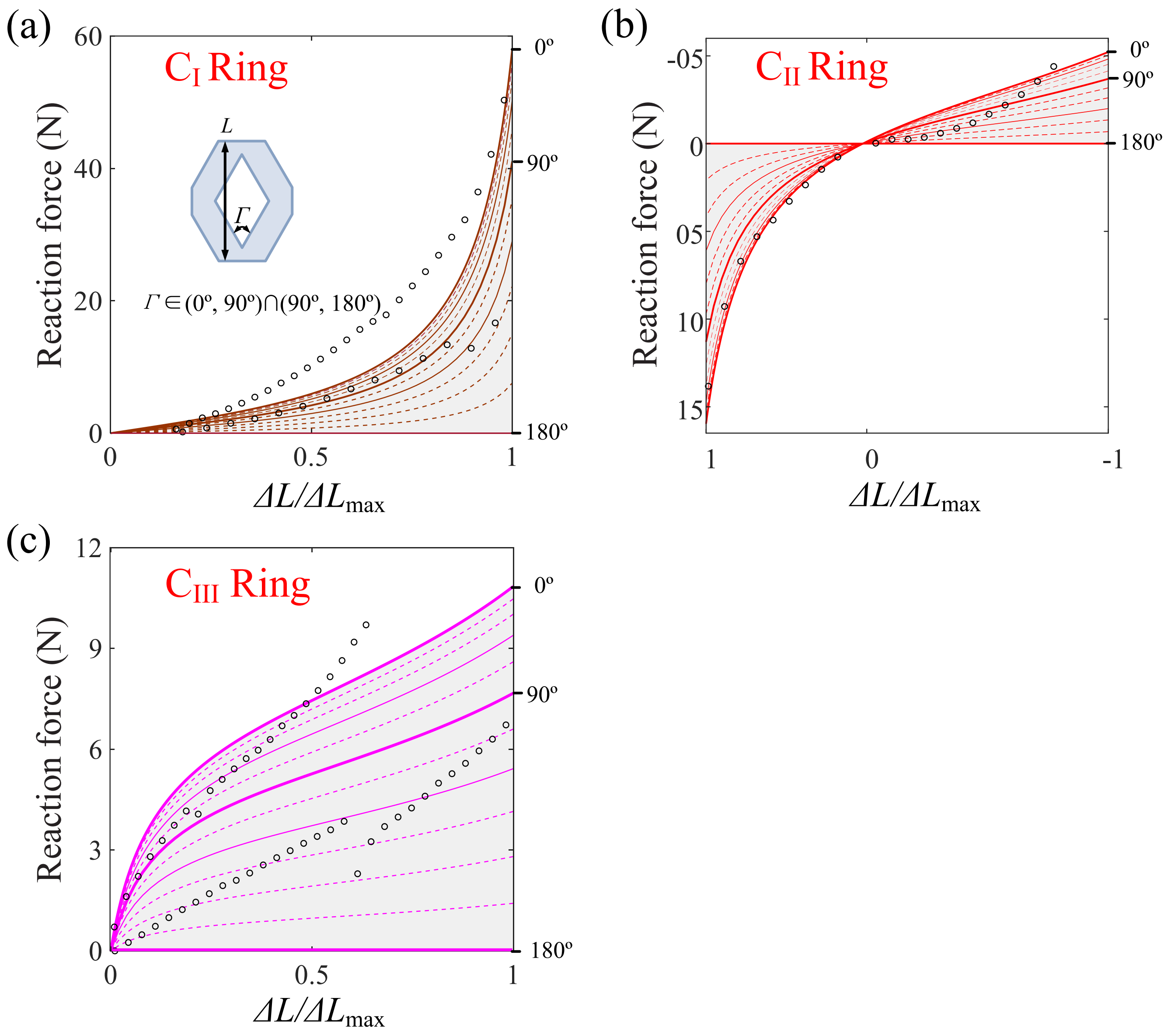

Supplementary Fig. 36: **The continuous reprogrammable force-displacement curve of C metamaterials of reprogrammable mechanical metamaterial and the experimental results.** (a) C metamaterial with C_I functional-groups. (b) C metamaterial with C_{II} functional-groups. (c) C metamaterial with C_{III} functional-groups. Source data are provided as a Source Data file.

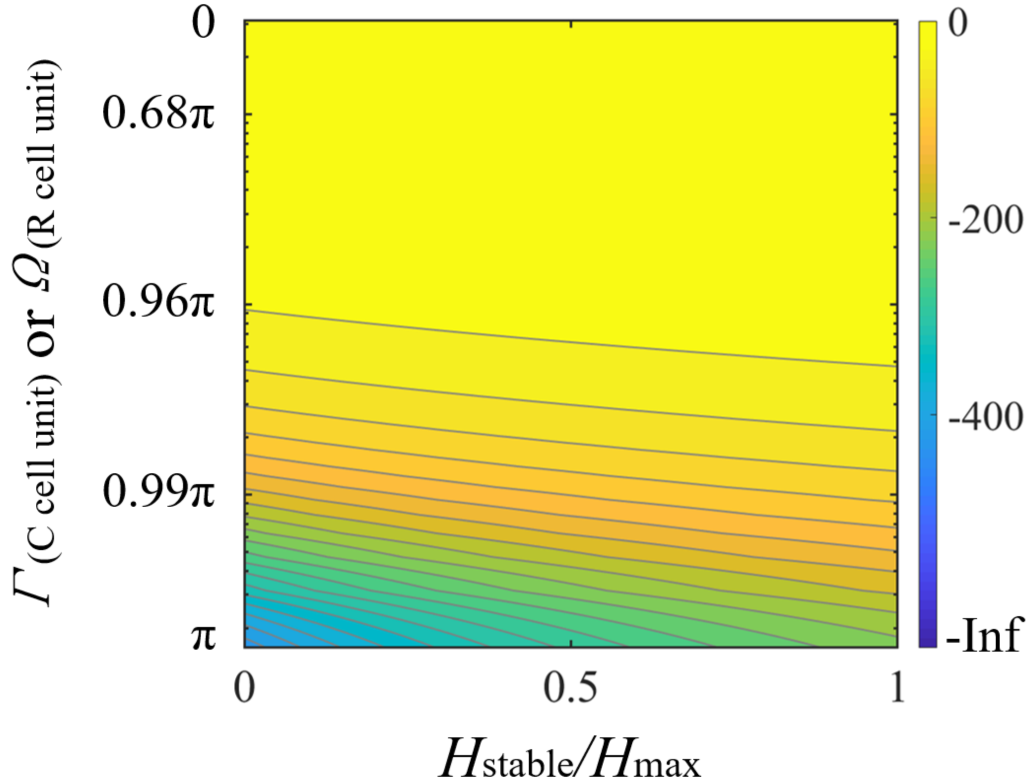

Supplementary Fig. 37: **The continuous reprogrammable Poisson's ratio of reprogrammable mechanical metamaterial.**

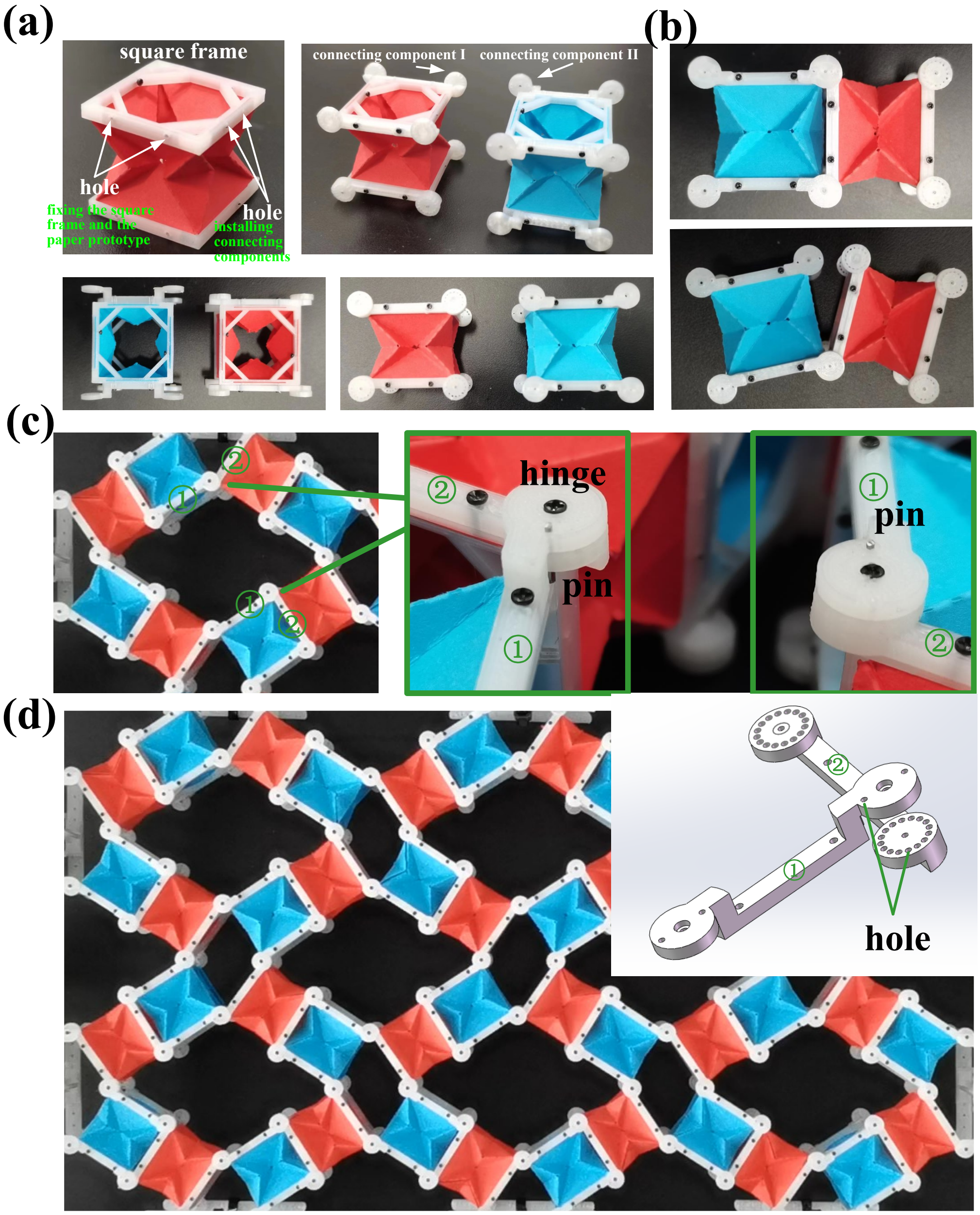

Supplementary Fig. 38: **The fabiraction of periodic homogeneous metamaterials with quadrilateral rings.** (a) The fabiracted paper prototype of origami elements equipped with square frames, connecting components and their presentations from different perspectives. (b) The fabiracted prototype of complete elastic functional-group. (c) The fabiracted prototype of complete elastic ring metamaterial, and the implementation method of angle adjustment and fixation under deformation. (d) The fabiracted of periodic homogeneous metamaterials with quadrilateral rings.

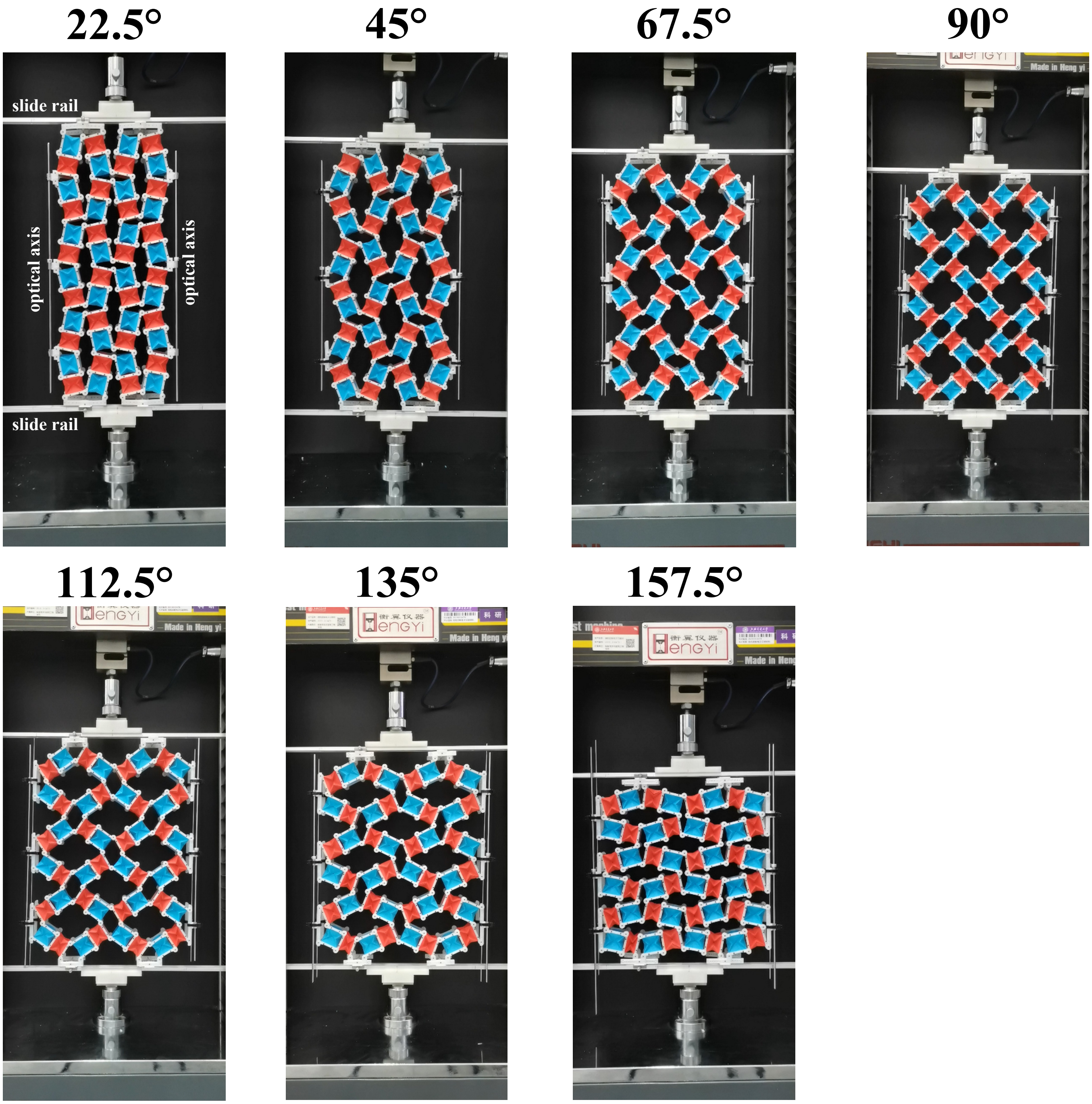

Supplementary Fig. 39: **Uniaxial mechanical tests of auxeticity under compression for periodic homogeneous metamaterials with** C_{III} **rings.** The adjusting angle is fixed with 22.5°, 45°, 67.5°, 90°, 112.5°, 135° and 157.5°.

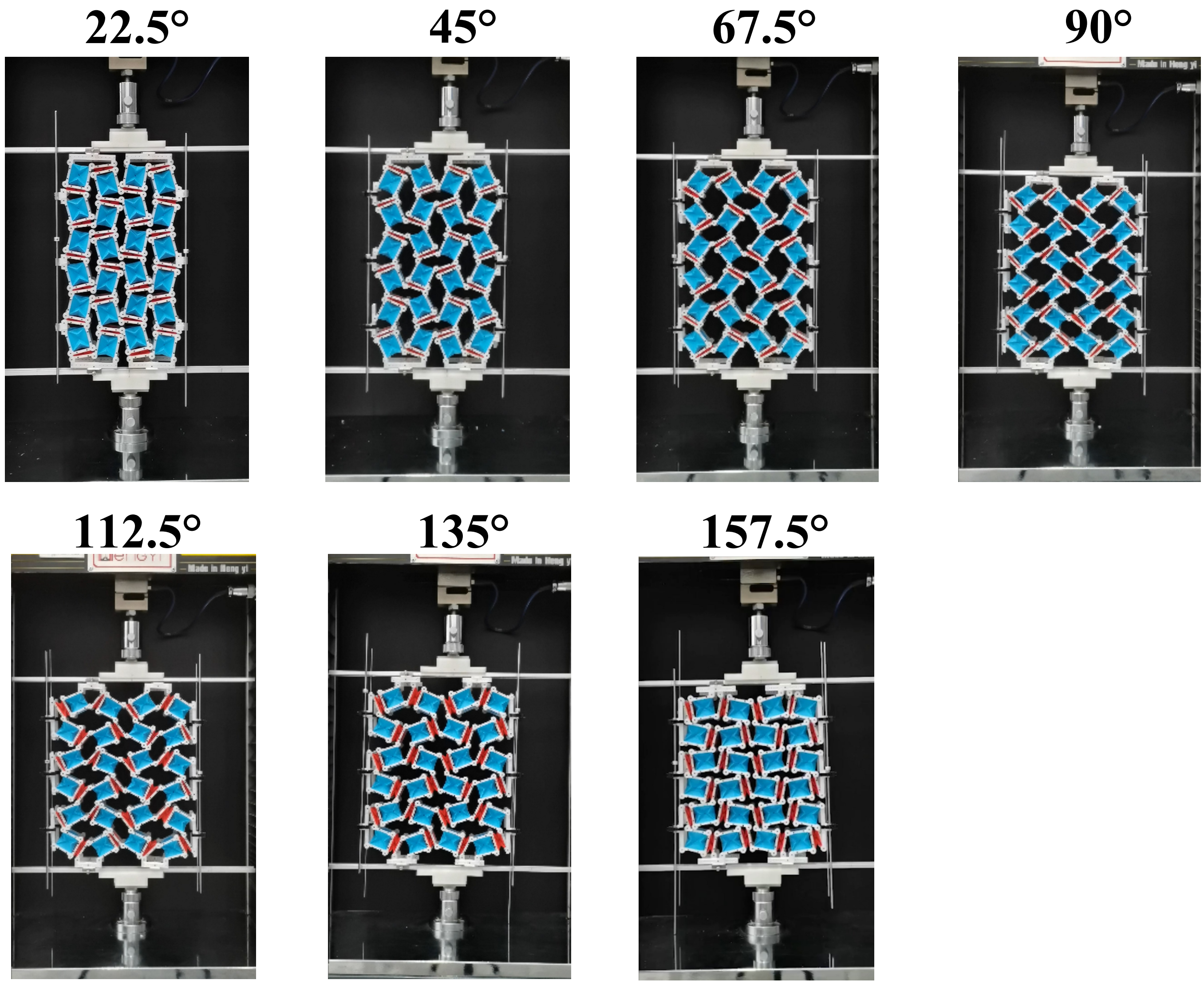

Supplementary Fig. 40: **Uniaxial mechanical tests of auxeticity under tension for periodic homogeneous metamaterials with** $\mathrm{C_I}$ **rings.** The adjusting angle is fixed with 22.5°, 45°, 67.5°, 90°, 112.5°, 135° and 157.5°.

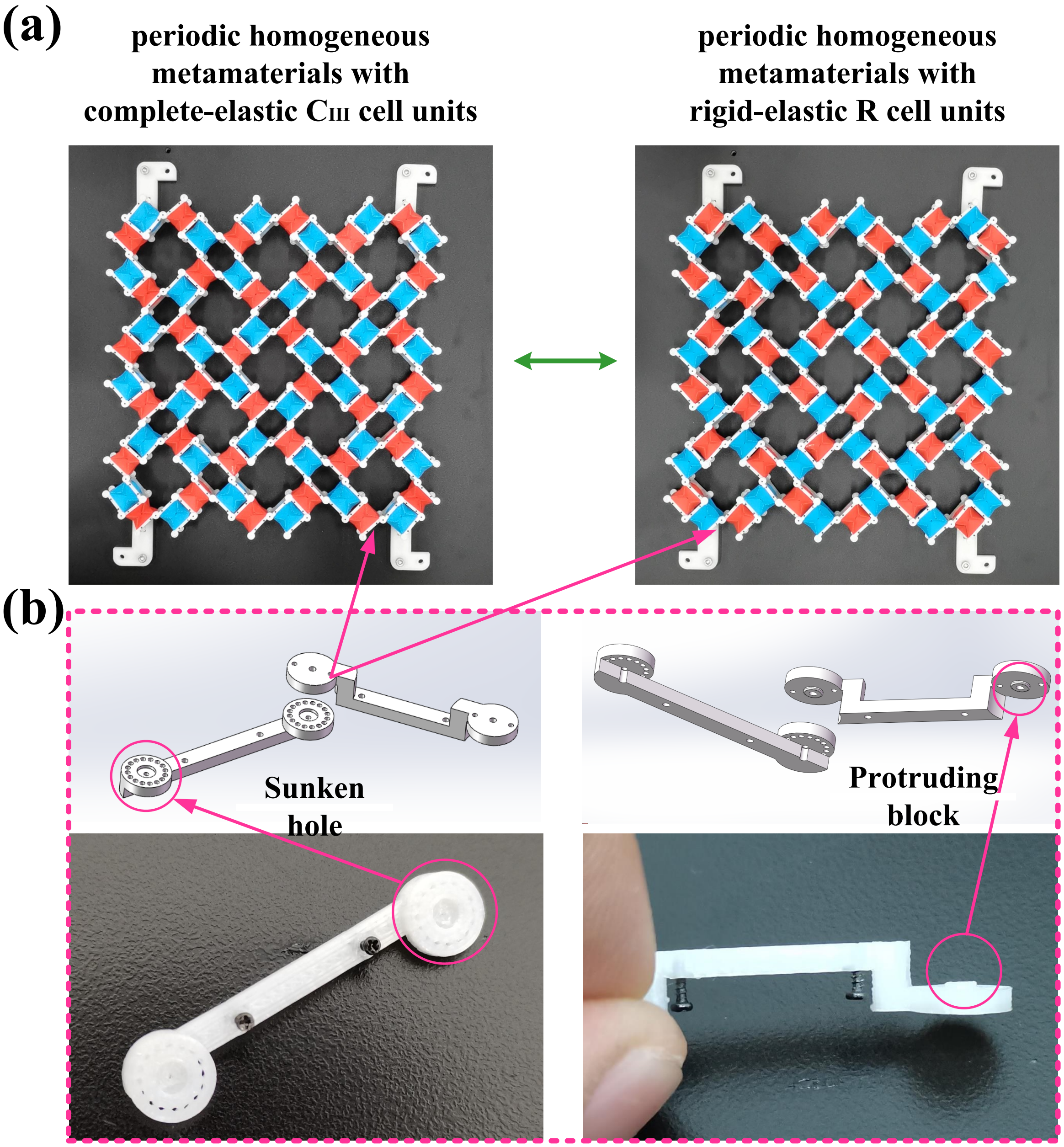

Supplementary Fig. 41: **The periodic homogeneous metamaterials with** $\mathrm{C_{III}}$ **cell units and transformed one with R cell units.** (a) The periodic homogeneous metamaterials with $\mathrm{C_{III}}$ and R cell units. (b) The connecting components of periodic homogeneous metamaterials between origami elements. In order to improve the transformation efficiency of Metamaterial, the connecting comopents are provided with sunken holes and protruding blocks that can be fastened with each other to replace the fixation of screws.

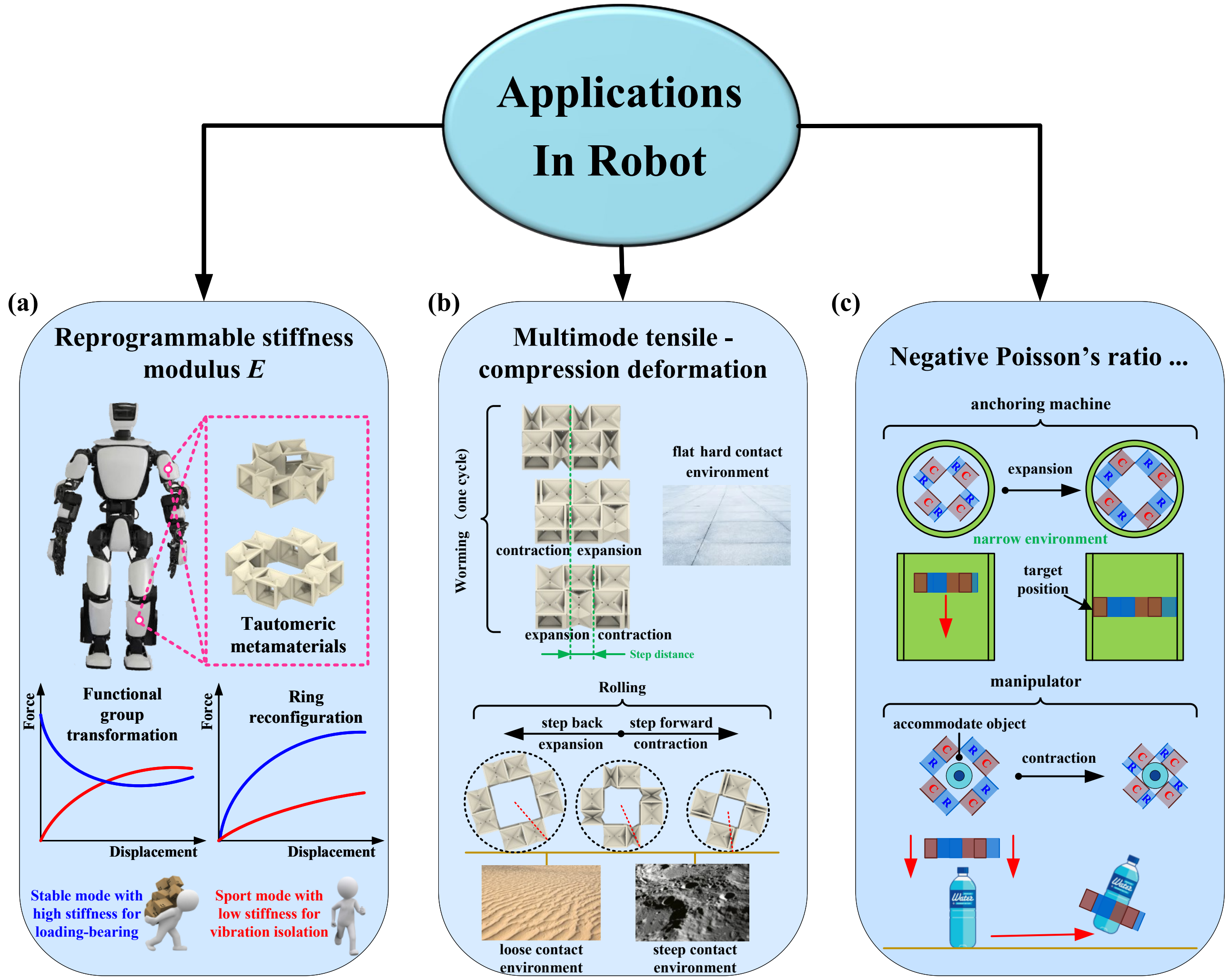

Supplementary Fig. 42: **Schematic diagram for applicationas of reprogrammable mechanical metamaterials in robot.** (a) body construction, such as legs and arms. (b) woriming and rolling actuator. (c) anchoring machine and manipulator.

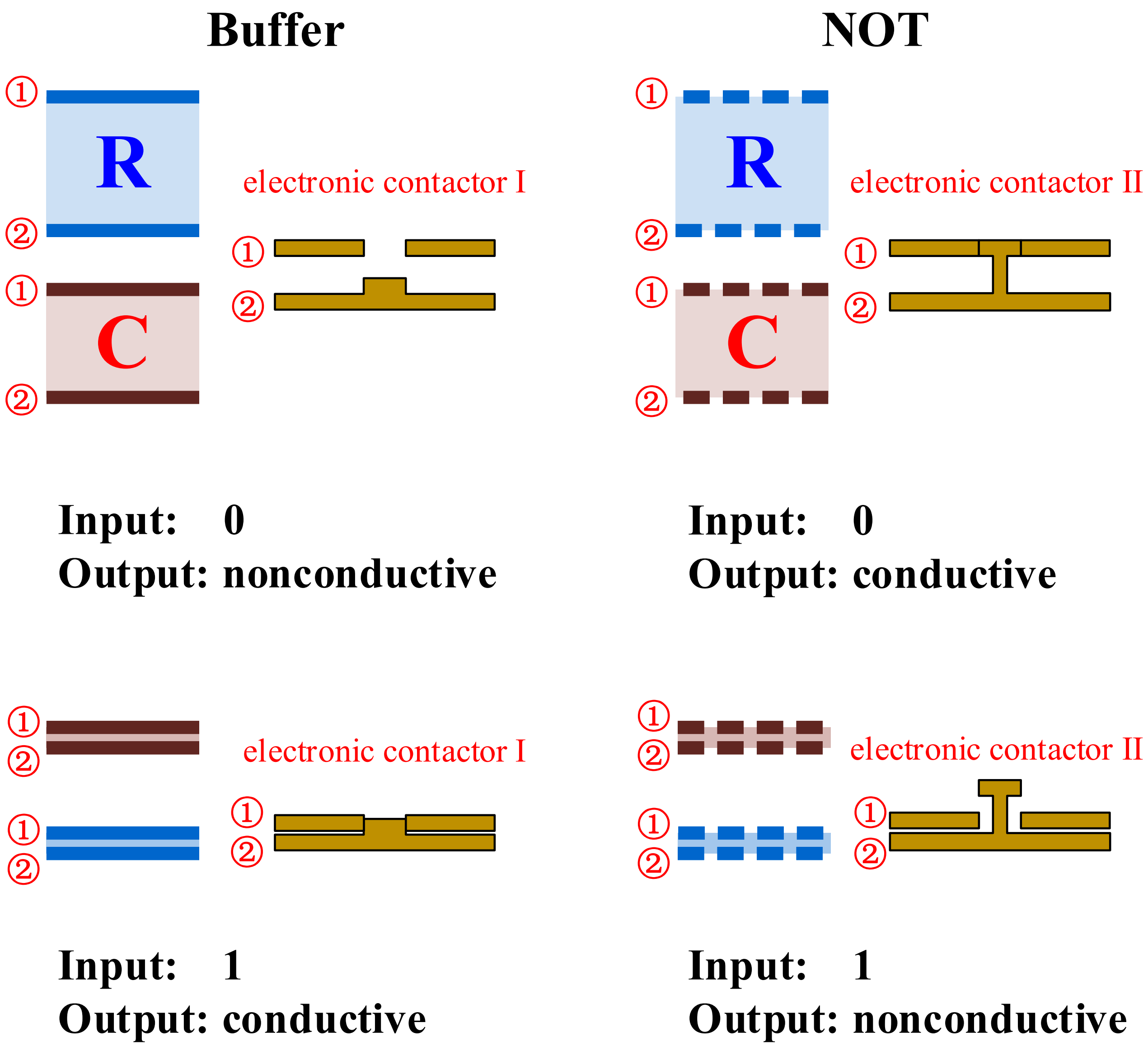

Supplementary Fig. 43: **Schematic diagram of Buffer, NOT logic gates and special electronic contacter I, II for realizing logic operation.**

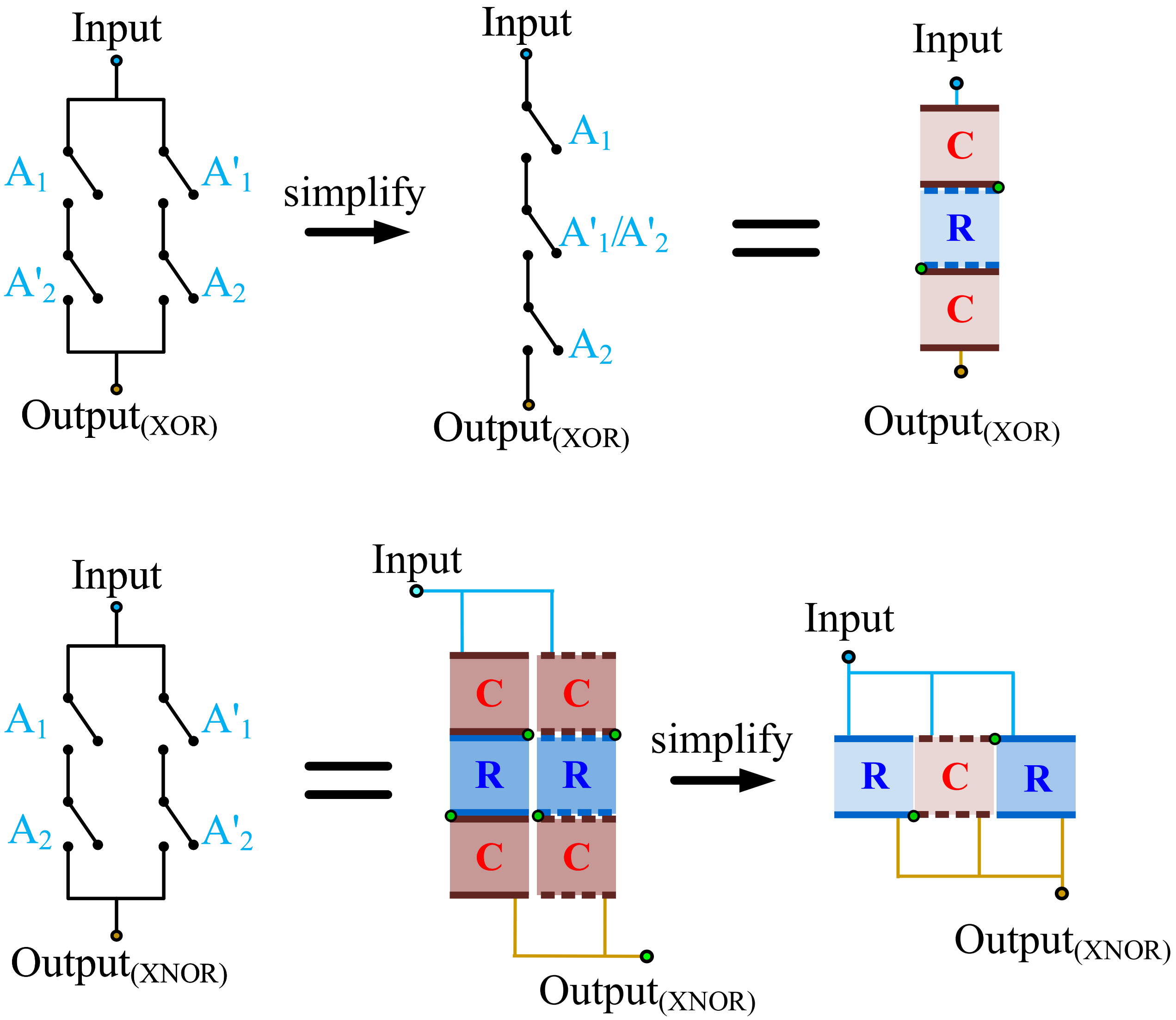

Supplementary Fig. 44: The Boolean operation diagram of XOR logic gate and XNOR logic gate and simplified mechanical structures based on the principle of simultaneous input (0,1) and (1,0).

Supplementary References

[1] Thomas, H. Project Origami: Activities for Exploring Mathematics. (CRC press, Boca Raton, Fla. 2006).

[2] . Hanna, B. H., et al. Waterbomb base: A symmetric single-vertex bistable origami mechanism. *Smart Materials and Structures* **23**, 094009 (2014).

[3] Boresi, A.P., Schmidt, R.J., Rega, G. Engineering mechanics: statics. Applied Mechanics Reviews. (CRC press, Boca Raton, Fla. 2002).

[4] Truszkiewicz E, Thalhamer A, Rossegger M, et al. Mechanical behavior of 3D-printed polymeric metamaterials for lightweight applications. *Journal of Applied Polymer Science* **139**, 51618 (2022).

[5] Mizzi L, Spaggiari A. Lightweight mechanical metamaterials designed using hierarchical truss elements. *Smart Materials and Structures* **29**, 105036 (2020).

[6] Wan M., Yu K., Sun H. 4D printed programmable auxetic metamaterials with shape memory effects, *Composite Structures* **279**, 114791 (2022).

[7] Bower A F. Applied mechanics of solids. (CRC press, Boca Raton, Fla. 2009).

[8] Agarwal B D, & Broutman L J. Analysis and performance of fiber composites, Second edition. (John Wiley & Sons, Hoboken, NJ, 1990).
