# Supplementary material for: A reprogrammable mechanical metamaterial with origami functional-group transformation and ring reconfiguration
Source: Nat Commun. 2023 Oct 23;14:6709. doi: 10.1038/s41467-023-42323-1 (PMC10593812; doi:10.1038/s41467-023-42323-1)
Supplement: Supplementary file 3 — Description of Additional Supplementary Files [file 41467_2023_42323_MOESM3_ESM.pdf]

Description of Additional Supplementary Files

File Name: Supplementary Movie 1
Description: Mechanical contribution of crease segments.

File Name: Supplementary Movie 2
Description: Deformation of origami element.

File Name: Supplementary Movie 3
Description: The transformation of functionalgroups and their deformation.

File Name: Supplementary Movie 4
Description: Torsional deformation of reprogrammable mechanical metamaterials.

File Name: Supplementary Movie 5
Description: Functional-group transformation of reprogrammable triangular ring metamaterials.

File Name: Supplementary Movie 6
Description: Transformation and ring reconfiguration of reprogrammable quadrilateral ring metamaterials.

File Name: Supplementary Movie 7
Description: Axial deformation of reprogrammable quadrilateral ring metamaterials.

File Name: Supplementary Movie 8
Description: Axial deformation of reprogrammable mechanical metamaterials in evolutionary morphologys.

File Name: Supplementary Movie 9
Description: Assembly of origami element.

File Name: Supplementary Movie 10
Description: Auxetic behaviour of periodic homogeneous metamaterials under compression.

File Name: Supplementary Movie 11
Description: Auxetic behaviour of periodic homogeneous metamaterials under tension.

File Name: Supplementary Movie 12
Description: Transformation of periodic homogeneous metamaterials.
